# BRD9-SMAD2/3 Orchestrates Stemness and Tumorigenesis in Pancreatic Ductal Adenocarcinoma

**DOI:** 10.1053/j.gastro.2023.09.021

**Published:** 2023-09-21

**Authors:** Yuliang Feng, Liuyang Cai, Martin Pook, Feng Liu, Chao-Hui Chang, Mai Abdel Mouti, Reshma Nibhani, Stefania Militi, James Dunford, Martin Philpott, Yanbo Fan, Guo-Chang Fan, Qi Liu, Jun Qi, Cheng Wang, Wanzi Hong, Hannah Morgan, Mingyang Wang, Sakthivel Sadayappan, Anil G. Jegga, Udo Oppermann, Yigang Wang, Wei Huang, Lei Jiang, Siim Pauklin

**Affiliations:** 1 Botnar Research Centre, Nuffield Department of Orthopaedics, Rheumatology and Musculoskeletal Sciences, University of Oxford, Oxford, United Kingdom; 2 Department of Pharmacology, School of Medicine, Southern University of Science and Technology, Guangdong, China; 3 Department of Cancer Biology, University of Cincinnati College of Medicine, Cincinnati, Ohio; 4 Department of Internal Medicine, Division of Cardiovascular Health and Disease, University of Cincinnati College of Medicine, Cincinnati, Ohio; 5 Departments of Pharmacology and Systems Physiology, University of Cincinnati College of Medicine, Cincinnati, Ohio; 6 Department of Cancer Biology, Dana-Farber Cancer Institute, Boston, Massachusetts; 7 Smurfit Institute of Genetics, Trinity College Dublin, Dublin, Ireland; 8 Department of Cardiology, Guangdong Cardiovascular Institute, Guangdong Provincial People’s Hospital (Guangdong Academy of Medical Sciences), Southern Medical University, China; 9 Heart, Lung and Vascular Institute, Department of Internal Medicine, Division of Cardiovascular Health and Disease, University of Cincinnati, Cincinnati, Ohio; 10 College of Engineering and Applied Science, University of Cincinnati, Cincinnati, Ohio; 11 Division of Biomedical Informatics, Cincinnati Children’s Hospital Medical Center, Cincinnati, Ohio; 12 Department of Pediatrics, University of Cincinnati College of Medicine, Cincinnati, Ohio; 13 Department of Computer Science, University of Cincinnati College of Engineering, Cincinnati, Ohio; 14 Oxford Translational Myeloma Centre, Botnar Research Centre, Oxford, United Kingdom; 15 Department of Pathology and Laboratory Medicine, University of Cincinnati College of Medicine, Cincinnati, Ohio

**Keywords:** Cancer Stem Cells, Cancer Therapy, Epigenetics, Pancreatic Cancer, TGF*β*/Activin-SMAD2/3

## Abstract

**BACKGROUND & AIMS:**

The dismal prognosis of pancreatic ductal adenocarcinoma (PDAC) is linked to the presence of pancreatic cancer stem-like cells (CSCs) that respond poorly to current chemotherapy regimens. The epigenetic mechanisms regulating CSCs are currently insufficiently understood, which hampers the development of novel strategies for eliminating CSCs.

**METHODS:**

By small molecule compound screening targeting 142 epigenetic enzymes, we identified that bromodomain-containing protein BRD9, a component of the BAF histone remodeling complex, is a key chromatin regulator to orchestrate the stemness of pancreatic CSCs via cooperating with the TGF*β*/Activin-SMAD2/3 signaling pathway.

**RESULTS:**

Inhibition and genetic ablation of BRD9 block the self-renewal, cell cycle entry into G0 phase and invasiveness of CSCs, and improve the sensitivity of CSCs to gemcitabine treatment. In addition, pharmacological inhibition of BRD9 significantly reduced the tumorigenesis in patient-derived xenografts mouse models and eliminated CSCs in tumors from pancreatic cancer patients. Mechanistically, inhibition of BRD9 disrupts enhancer-promoter looping and transcription of stemness genes in CSCs.

**CONCLUSIONS:**

Collectively, the data suggest BRD9 as a novel therapeutic target for PDAC treatment via modulation of CSC stemness.

## Graphical Abstract

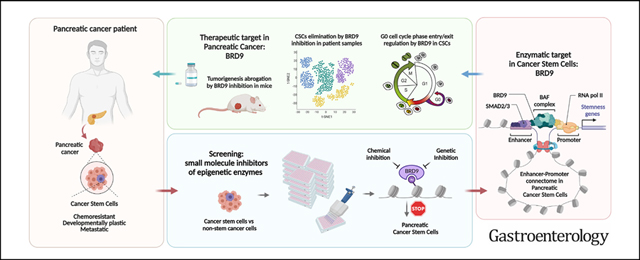

Pancreatic cancer with its most common type, pancreatic ductal adenocarcinoma (PDAC), is one of the most lethal human malignancies.^1–3^ It has an overall median survival time of 6 to 9 months and a similarly 5-year survival rate of 6%, making it currently the fourth leading cause of cancer-related deaths in western countries.^4,5^ Because of the increasing incidence of risk factors, including obesity and other metabolic traits, pancreatic cancer is projected to overtake colorectal, breast, and prostate cancer and become the second leading cause of worldwide cancer-related deaths by 2030.^6^ The disease owes its exceptional level of lethality to multiple factors. Pancreatic cancer often has no early symptoms and presents itself in an advanced stage at diagnosis: only 20% of newly diagnosed pancreatic cancers are amenable to surgery.^7^ In turn, the disease’s poor response to chemotherapy and radiotherapy results in disease reemergence for 90% of the surgically treated patients.^8^ Treatment options for PDAC are limited and inefficient. Gemcitabine, an antimetabolite drug of the nucleoside analogue class is the standard of care in PDAC therapy.^8^ It is currently the only approved single-agent drug for pancreatic cancer, with still modest improvements in survival rate,^9^ whether the drug is administered alone or in combination with adjuvant drugs such as the epidermal growth factor receptor inhibitor erlotinib^10^ or the tubulin-targeting drug Nab-paclitaxel.^9^ A combination therapy such as FOLFIRINOX is superior to gemcitabine-based regimens in restraining the progression of metastatic PDAC but has lower tolerability.^11^

Precancerous lesions and dedifferentiation of the cells to a progenitor-like or stem cell–like state with increased cellular plasticity frequently occur during pancreatic tissue transformation.^4,12^ A distinct cell population often referred to as cancer stem cells (CSCs), seems to acquire a stem cell–like state partially resembling naturally occurring stem cells.^13–15^ This phenotype allows them to give rise to the whole tumor with its entire cellular heterogeneity and thereby supports metastases formation and development of resistance to current cancer therapeutics. The existence of developmentally plastic CSCs has been discovered in the brain, breast, colon, esophagus, liver, lung, ovarian, prostate, stomach, and thyroid cancers, among others. In the case of PDAC, the first reports of CSCs date back to 2007.^13,14^ Since then, pancreatic CSCs have been conclusively shown to be involved in PDAC resistance to chemotherapy, displaying increased prevalence within the tumor after treatment with gemcitabine.^16,17^ Annihilating CSCs is thus emerging as an essential aim of PDAC therapeutics. CSCs are thought to have specific epigenetic mechanisms^18^ that regulate their self-renewal, and the formation of CSCs has been postulated to occur as a result of epigenetic events.^19^ Accordingly, cancer epigenetics has established itself as a promising area of oncology research.^20^ After a decade in which epigenetic cancer drugs were approved only for hematological malignancies, the first Food and Drug Administration approval for an epigenetic drug targeting solid tumors was granted in 2020 for an EZH2 inhibitor. Despite their paradigm-shifting novel mechanism of action, early results of epigenetic modulators have identified the need for better selection of targets, improved intratumoral drug penetration, and elimination of CSCs.

Pancreatic cancers are complex tumors with significant heterogeneity in their molecular and cellular make-up that are controlled by various signaling pathways that crosstalk with epigenetic regulators. Among these pathways is the transforming growth factor (TGF)*β*/Activin/Nodal-SMAD2/3 pathway. This developmental signaling pathway plays a central role in early development by regulating the self-renewal of human pluripotent stem cells, epithelial-to-mesenchymal transition (EMT), and pancreatic tissue homeostasis.^21–23^ The TGFß/Activin/Nodal-SMAD2/3 pathway regulates epigenetic mechanisms, for instance by cooperating with the core pluripotency protein NANOG and epigenetic modifiers such as DPY30–complex of proteins associated with Set 1 (COMPASS) to control pluripotency and differentiation of human pluripotent stem cells.^24^ Aside from the key role of TGF*β*/Activin/Nodal-SMAD2/3 in pluripotent stem cells and developmental processes, this signaling pathway is directly involved in the formation of PDAC^22^ and is frequently deregulated in PDAC.^25,26^ The function of the pathway in PDAC is particularly interesting, because it confers dedifferentiated stem cell–like features to CSCs in PDAC,^15^ although the underlying mechanisms are still largely unknown.

Our hypothesis was that epigenetic or chromatin-templated mechanisms are essential to main CSC properties and that inhibition of these mechanisms may provide a path forward to modulate CSC phenotypes. Using a focused compound library of epigenetic inhibitors, we performed a small molecule compound screening and identified the BAF chromatin complex component BRD9 as a critical regulator of CSC behavior. Our results indicate that BRD9 is an attractive therapeutic target for specifically eliminating CSCs in PDACs.

## Materials and Methods

For full materials and methods and the full list of references, please see the accompanying supplementary information.

## Results

### Development of a Screening Platform to Target PDAC CSCs

**Figure 1.**
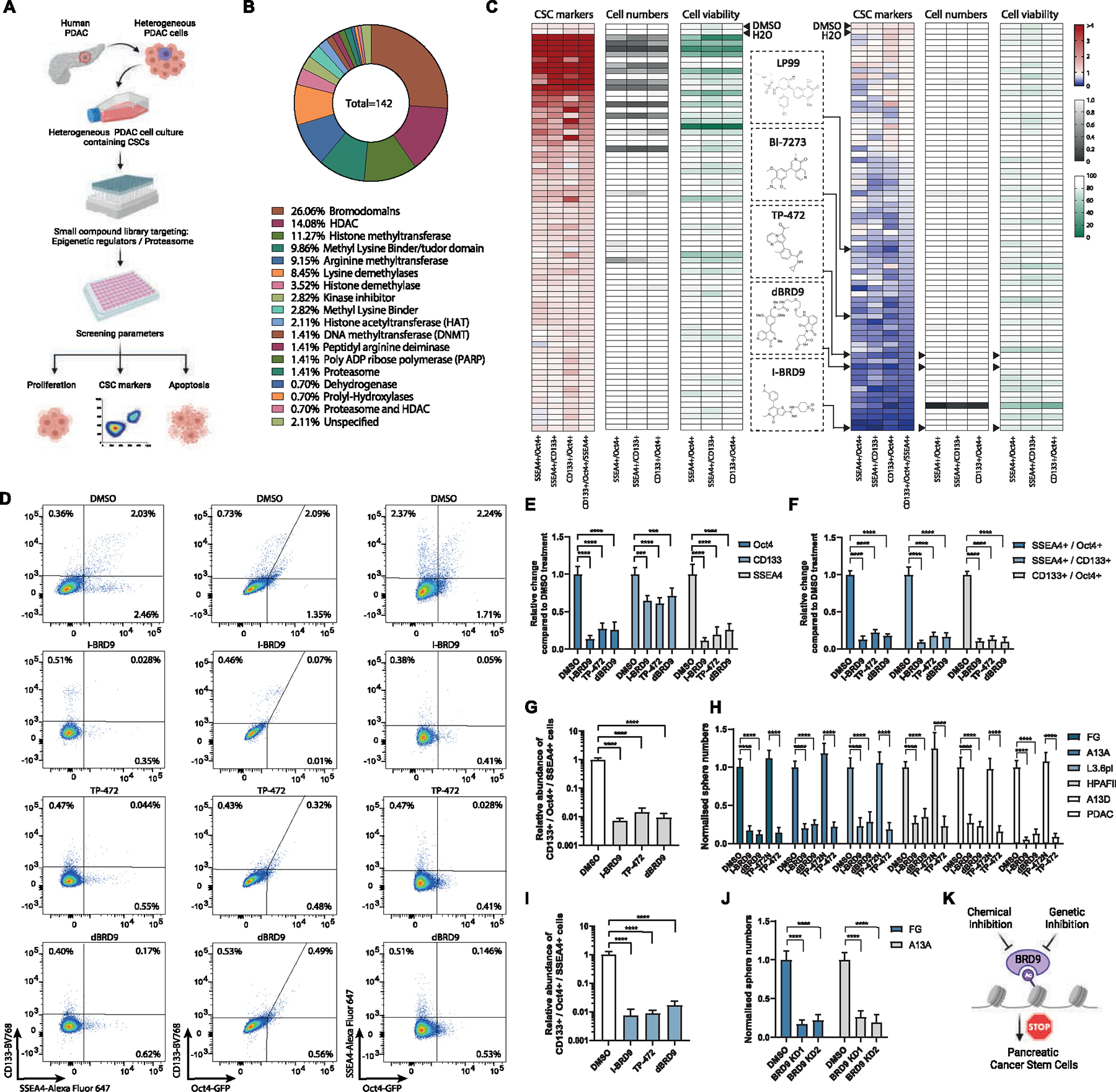
Chemical screening identifies BRD9 as a regulator of pancreatic CSCs. (*A*) Schematic depiction of the small molecule compound screening process on pancreatic cancer cells. (*B*) The classification of the compound library based on the percentage of the 142 compounds belonging to each class of enzymes they target. (*C*) BRD9 inhibitors decrease the relative number of cells that express CSC markers OCT4-GFP, CD133, and SSEA4 as double-positive cells or triple-positive cells. Heat maps of chemical screening depicting the relative change in the expression of CSC markers, cell numbers, and cell viability. (*D*) BRD9 inhibitors reduce the percentage of OCT4-GFP, CD133, and SSEA4 double-positive cells. (*E–G*) BRD9 chemical inhibitors reduce OCT4-GFP, CD133, and SSEA4 marker expression in pancreatic cancer cells. The relative change in single CSC marker-positive (*E*), double-positive (*F*), and triple-positive cells compared with control DMSO treatments (*G*). (*H*) BRD9 inhibition reduces CSC self-renewal in different PDAC cell lines and PDAC cells from surgically resected tumor. (*I*) The relative decrease in OCT4-GFP+/CD133+/SSEA4+ cancer cells by BRD9 inhibition in L3.6pl PDAC cell line. (*J*) BRD9 KD reduces CSC self-renewal in PDAC cell lines. (*K*) Schematic depiction of the effects of BRD9 inhibition in CSCs. Experiments represent 3 replicates. Statistical analysis was performed by 2-way analysis of variance with multiple comparisons with Tukey correction. ****Adjusted *P* < .0001, ***adjusted *P* < .001, **adjusted *P* < .01, *adjusted *P* < .05.

Because PDAC cells comprise a heterogeneous population of CSCs and non-CSCs, we first decided to establish a suitable small molecule screening platform in PDAC cells (Figure 1A). To uncover novel regulators of CSCs, we used 3 CSC markers (OCT4, CD133, and SSEA4)^27–30^ for identifying stem cell–like cells in the experiments. The FG pancreatic cancer cells used for this screening were genetically engineered, with the sequence coding for enhanced green fluorescent protein (eGFP) integrated in the endogenous locus via TALEN-mediated recombination, resulting in controlled expression of a OCT4-eGFP fusion protein driven by the endogenous *OCT4* promoter (Supplementary Figure 1A). To validate the importance of CD133, SSEA4, and OCT4 as markers for the CSC population in our PDAC cells, we treated the cells with chemotherapy reagents gemcitabine, paclitaxel, and 5-fluorouracil that are currently in clinical use as PDAC patient therapeutics. Gemcitabine, paclitaxel, and 5-fluorouracil treatment of PDAC cells for 5 days enriched for cells expressing OCT4-GFP, CD133, and SSEA4 (Supplementary Figure 1B), by eliminating non-CSCs (Supplementary Figure 1C). This results in selective survival and enrichment of the rare OCT4-GFP+/CD133+/SSEA4+ CSCs.

Because the TGF*β*/Activin/Nodal-SMAD2/3 developmental signaling pathway regulates pluripotency via epigenetic regulatory complexes and impacts stem cell–like characteristics of CSCs,^24,31^ we also tested the impact of TGF*β*/Activin signaling on CSC resistance to currently used chemotherapeutics by treating the cells with the TGF*β*/Activin signaling inhibitor SB431542 in combination with gemcitabine, paclitaxel and 5-fluorouracil (Supplementary Figure 1B and C). Inhibition of TGF*β*/Activin signaling strikingly reduced the chemoresistance of PDAC cells as indicated by reduced numbers of OCT4-GFP+/CD133+/SSEA4+ CSCs (Supplementary Figure 1B) and therefore the overall number of surviving PDAC cells (Supplementary Figure 1C). These results emphasize the crucial importance of TGF*β*/Activin signaling on CSC maintenance and their elevated chemoresistant characteristics. Furthermore, patients with PDAC with high expression of CD133 and OCT4 have lower overall survival (Supplementary Figure 1D) and lower disease-free survival (Supplementary Figure 1E). CD133+/OCT4+/SSEA4+ cells consistently showed higher CSC sphere formation capacity compared with unsorted cells, whereas CD133−/OCT4−/SSEA4− had lower self-renewal capacity compared with unsorted cells and CD133+/OCT4+/SSEA4+ cells (Supplementary Figure 1F). In addition, a 2-fold limiting dilution assay with CD133+/Oct4+/SSEA4+ cells from FG line compared with unsorted cells indicated a significantly elevated self-renewal capacity (Supplementary Figure 1G).

We also investigated the effect of gemcitabine on CSCs. We dissociated primary human PDAC tissue from patients into single cells and treated these with gemcitabine for 72 hours followed by flow cytometry analyses of CSC markers. The expression of CSC markers CD133, OCT4, and SSEA4 on live PDAC cells indicated an enrichment of the CSC markers on gemcitabine treatment compared with control dimethyl sulfoxide (DMSO) treatment (Supplementary Figure 1H–J). This is in agreement with studies showing that gemcitabine treatment increases CSC enrichment, and supports our experimental data in PDAC cell lines, which showed the enrichment of CSCs on gemcitabine treatment.

Collectively, these results underline the suitability of our screening platform and the translational relevancy of this preclinical model for compound screening.

### A Focused Compound Library Screen of Epigenetic Inhibitors Identifies BRD9 as an Important Factor for Governing CSC Characteristics

Because of the importance of epigenetic pathways in tumorigenesis, we hypothesized that the formation and maintenance of pancreatic CSCs are controlled by epigenetic mechanisms, and these could be used as therapeutic targets for eliminating CSCs. To address this, we performed a focused library screen consisting of validated small molecule inhibitors (Supplementary Table 1) targeting epigenetic regulators such as “readers, writers, and erasers” of a histone code.^32^ These experiments aimed to identify molecular targets of small molecule compounds that specifically affect CSC marker-expressing cells (Figure 1A–C). We measured CSC marker expression, cell growth, and cell death by flow cytometry (CD133+/OCT4+/SSEA4+) after incubating the cells with the compounds for 5 days, which allowed us to identify effective compounds that affect CSC marker-expressing populations while also detecting cells that do not express these CSC markers. The compound library used in our experiments consisted of 142 compounds that have been verified to be active and targeting specific epigenetic modifying enzymes (Figure 1C). Overall, this screening identified compounds that significantly and reliably reduced the relative percentage of triple+ marker (CD133+/OCT4+/SSEA4+) expressing CSCs and also reduced cancer cell survival (Figure 1C). Our study identified the BET bromodomain inhibitors I-BET 672 and JQ1,^33,34^ confirming the suitability of our screening platform.

Importantly, the screening identified novel compounds that target distinct epigenetic regulatory components and strikingly reduced the percentage of CSCs. Among the top candidate compounds with a distinct effect on CSCs, we identified BRD9 inhibitors (I-BRD9, dBRD9, TP-472, BI-7273, LP99) (Figure 1C and D). These results suggested that BRD9 inhibition could induce differentiation of therapy-resistant CSCs into a more therapy-responsive population and thus could possibly sensitize PDAC cancers to conventional therapy. BRD9 is a component of the noncanonical BAF chromatin remodeling complex (ncBAF) relevant for stem cells and tumorigenesis,^35–37^ leading us to focus our investigation on the molecular role of the BAF complex and BRD9 in particular, in PDAC CSCs.

To further validate BRD9 as a target, we used 2 BRD9 chemical inhibitors (I-BRD9, TP-472) and the PRO-TAC degrader of BRD9 (dBRD9)^38^ to investigate their effects on CSC markers (Supplementary Figure 1K and L). All 3 inhibitors of BRD9 resulted in the reduction of OCT4, CD133, or SSEA4 single marker-positive cells (Figure 1E), even more pronounced reduction in double-positive cells for OCT4+/SSEA4+, CD133+/SSEA4+, or CD133+/OCT4+ (Figure 1F), and with a most striking reduction in OCT4+/CD133+/SSEA4+ triple-positive PDAC cells (Figure 1G). I-BRD9 and TP-472 reduced the OCT4+/CD133+/SSEA4+ CSC numbers also in other PDAC cell lines (Figure 1I).

The bulk cell population of the PDAC is sensitive to chemotherapy and the CSC subpopulation of PDAC cells is the reason why PDAC is therapy recalcitrant. Therefore, we investigated the effects of compounds on CSCs by 3-dimensional (3D) tumor sphere assays. To study the impact of BRD9 inhibition on CSC self-renewal capacity, we performed tumor sphere assays with 3 different BRD9 inhibitors (I-BRD9, TP-472, and dBRD9) in 5 different PDAC cell lines and primary cells from surgically resected PDAC tumors (Figure 1H). Inhibition of BRD9 with these 3 inhibitors showed a significant reduction in CSC sphere numbers. To investigate the effect of BRD9 inhibition by genetic means, we performed stable BRD9 knockdown (KD) in 2 PDAC cell lines (Supplementary Figure 1M–P). Tumor sphere assay revealed a reduction of spheres in both cell lines by 2 different short hairpin RNA (shRNA) constructs (Figure 1J), indicating that BRD9 loss-of-function via KD phenocopies the reduction of CSC self-renewal as seen with chemical inhibition of BRD9 (Figure 1K).

Based on our screening, BRD1, BRD7, BRD4, and SMARCA factors could potentially have a compensatory effect on maintaining CSCs on the inhibition of BRD9 (Supplementary Figure 1R and S). BRD1, BRD4, and SMARCA2/4 inhibition decreases CSC self-renewal shown by the decrease in CSC sphere numbers and on co-inhibition (Supplementary Figure 1T), suggesting that there are additional effects of BRD7, BRD1, BRD4, and SMARCA2/4 that do not fully overlap or compensate for the function of BRD9 in CSCs.

In PDAC, several nonoverlapping heterogeneous CSC populations have been identified that express markers such as CD133+, CD44+, ALDH+, CXCR4, DCLK1, and MDR1+. To investigate if BRD9 inhibition can also affect other nonoverlapping CSC populations, we used flow cytometry analyses of CSC marker expression in PDAC cell lines and PDAC patient-derived primary tumor samples. Flow cytometry analyses of 3 PDAC cell lines indicated a reduction of CD44−, CD133−, ADH1−, CXCR4−, DCLK1−, and MDR1− expressing cells on I-BRD9 treatment compared with control DMSO treatment (Supplementary Figure 2A and B). We also investigated the effects of I-BRD9 in patient-derived primary PDAC tumor samples depleted for CD45+ cells. We observed a decrease in the CSC marker-expressing cells (CD44, ALDH, CXCR4, DCLK1, and MDR1) on I-BRD9 treatment also in primary PDAC patient samples (Supplementary Figure 2C and D). Collectively, I-BRD9 treatment reduces the nonoverlapping CSC populations in pancreatic cancers.

### BRD9 Inhibition Reduces CSC Entry to G0 Cell Cycle Phase and Chemoresistance

**Figure 2.**
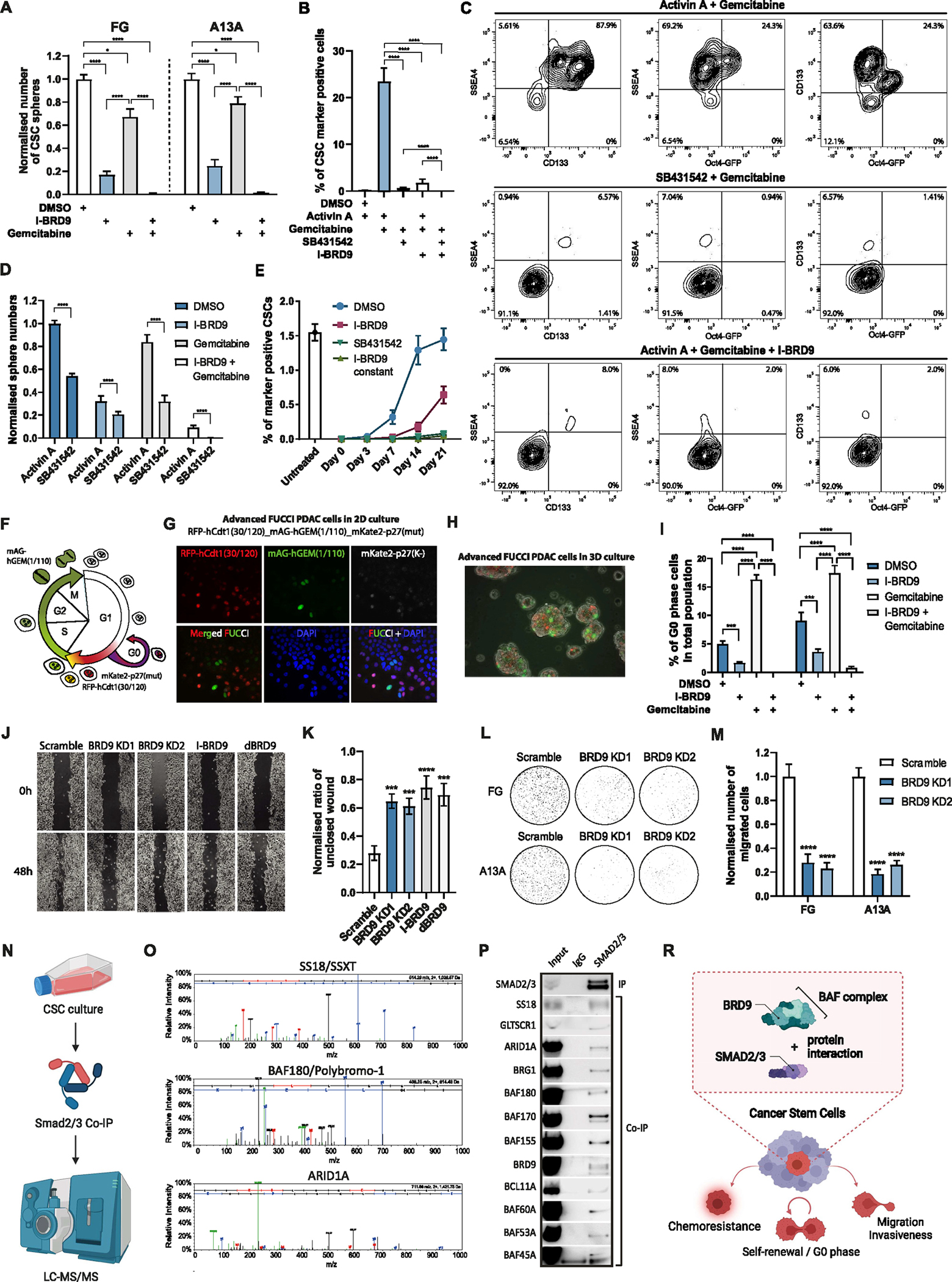
BRD9 inhibition abolishes CSC characteristics and blocks cells from entering the G0 phase. (*A*) BRD9 inhibition sensitizes CSCs for gemcitabine-mediated elimination. (*B* and *C*) Triple-positive OCT4−GFP+/CD133+/SSEA4+ CSC marker reduction in FG cells is induced by gemcitabine cotreatment with I-BRD9 or SB431542 as shown in bar graph (*B*) or density maps from flow cytometry analyses (*C*). (*D*) TGF*β*/Activin signaling inhibition and BRD9 inhibition impair CSC self-renewal and sensitize CSCs for gemcitabine-mediated destruction. (*E*) BRD9 inhibition shifts the balance between CSCs and non-CSCs toward non-CSCs. (*F*) Schematic depiction of the 3-color cell cycle analysis system. (*G*) Fluorescence microscopy of 3FUCCI-PDAC cells grown in 2D condition. (*H*) Fluorescence microscopy overlayed with bright field image of 3FUCCI-PDAC cells grown in 3D sphere condition. (*I*) BRD9 inhibition blocks cells from entering the G0 phase. (*J* and *K*) BRD9 inhibition reduces cell migration. (*J*) Normalised data of wound-healing assay as a bar graph. (*L* and *M*) BRD9 inhibition reduced PDAC cell invasiveness. (*N*) Schematic overview of the SMAD2/3 proteomic experiment. (*O*) SS18/SSXT, BAF180/Polybromo-1, and ARID1A form a complex with SMAD2/3 in CSCs. (*P*) SMAD2/3 proteins interact with the subunits of noncanonical BAF, esBAF, and npBAF complexes in CSCs. (*R*) TGF*β*/Activin A signaling leads to the formation of protein complex between SMAD2/3 and BRD9/BAF that regulates the function of chemoresistance, G0 phase entry, migration, and invasiveness of CSCs. Experiments represent 3 replicates. Statistical analysis was performed by 2-way analysis of variance with multiple comparisons with Tukey correction. ****Adjusted *P* < .0001, ***adjusted *P* < .001, **adjusted *P* < .01, *adjusted *P* < .05.

Because CSCs have lower sensitivity to chemotherapeutics besides their self-renewing capacity, we investigated the impact of BRD9 inhibition on the chemoresistance of CSCs in combination with gemcitabine treatment (Figure 2A). BRD9 inhibition together with gemcitabine treatment led to a stronger reduction in CSC sphere formation compared with either I-BRD9 or gemcitabine treatment alone, suggesting that BRD9 inhibition sensitizes CSCs for gemcitabine-mediated cell killing. BRD9 depletion/inhibition also moderately slowed cell proliferation in the 2-dimensional condition (Supplementary Figure 3A and B), and sensitized the bulk PDAC cells to chemotherapy (Supplementary Figure 3C). SMARCA2 and SMARCA4 depletion with PRO-TAC AU-15330 and BRM/BRG1 ATP Inhibitor-1 increased the sensitivity of PDAC cells to chemotherapy indicated by the reduced number of CSC spheres on gemcitabine treatment (Supplementary Figure 3D and E). Therefore, BAF/PBAF ATPase inhibition sensitizes CSCs to chemotherapy while also moderately reducing the bulk PDAC cells in response to gemcitabine.

BRD9 inhibition as well as TGF*β*/Activin pathway inhibition strikingly increased the sensitivity of CSCs to gemcitabine (Figure 2B and C; Supplementary Figure 3F and G), whereas the combination of BRD9 inhibition and gemcitabine treatment together with TGF*β*/Activin pathway inhibition very efficiently eliminated the formation of spheres (Figure 2B and D; Supplementary Figure 3H–K). Given the mutual exclusivity of BRD9 and BRD7 in the BAF and PBAF complexes, we also found a mechanistic balance between BAF and PBAF complexes in affecting the stemness of PDAC cells (Supplementary Figure 3L–O).

Because of the plasticity of the cell state, CSCs and nonstem cancer cells have been proposed to form a dynamic balance between differentiation and dedifferentiation. BRD9 inhibition could promote CSC differentiation or decrease cellular plasticity by increasing the epigenetic barriers that are necessary for the cells to dedifferentiate from nonstem cancer cells to CSC (Figure 2E). The fluorescent ubiquitination-based cell cycle indicator (FUCCI) system is a powerful tool to assess cell cycle–dependent responsiveness to drugs and the effect of drugs, gene silencing, or activation on the cell cycle without the need for synchronization.^3–43^ We developed a 3-color FUCCI system (RFP-hCdt1(30/120)_mAG-hGEM(1/110)_mKate2-p27(mut)) to distinguish between the cells in early G1, late G1, early S, S/G2/M, and G0 phases (Figure 2F–H). In PDAC cell lines, gemcitabine increased G0-phase cells, and I-BRD9 treatment led to a reduction in G0-phase cells and also blocked cells from accumulating in the G0-phase on gemcitabine treatment (Supplementary Figure 4A–D; Figure 2I). This suggested that BRD9 inhibition reduces the capacity of cells to enter the G0 phase and this could be particularly useful in eliminating CSCs on combination treatments with DNA damaging reagents, such as gemcitabine, that prevent the cells from escaping genotoxic insults through temporarily dormant or quiescent states by entering the G0 phase.

Wound-healing assays with BRD9 genetic knockdown, chemical inhibition, and protein degradation indicated reduced migration of PDAC cells compared with control cells (Figure 2J and *K*). Transwell migration assay by using BRD9 KD cells also showed a reduction in cell migration, thus supporting our conclusions from the wound-healing assays (Supplementary Figure 4E). Importantly, BRD9 KD also reduced PDAC cell invasiveness by transwell assays in 2 separate PDAC lines (Figure 2L and M), indicating that BRD9 inhibition could be useful for reducing the metastatic capacity of PDACs because BRD9 inhibition decreases PDAC cell motility and invasiveness.

Collectively, chemical inhibition and KD of BRD9 blocked the self-renewal of CSCs, reduced CSC invasiveness, and re-sensitized PDAC CSCs to conventional chemotherapeutic reagents, suggesting that BRD9 could be an attractive therapeutic target in PDAC.

### BRD9/BAF Complex Cooperates With SMAD2/3 in CSCs

We proceeded with identifying the binding partners of SMAD2/3 proteins in CSCs by performing SMAD2/3 co-immunoprecipitation followed by mass spectrometry (Figure 2N). This unbiased proteomic approach identified SS18/SSXT, BAF180/Polybromo-1, and ARID1A peptides, indicating that these proteins could be cofactor candidates of SMAD2/3 (Figure 2O). STRING analysis of protein interactions confirmed that these 3 proteins are all part of the ATP-dependent chromatin remodeling complex BAF (BRG1/BRM-associated factor) that corresponds to the mammalian SWI/SNF complex (Supplementary Figure 4F). The BAF complex has tissue-specific functions that arise from the combinatorial assembly of distinct subunits, whereas BRD9 protein is a common subunit.^44–46^ Therefore, we investigated further the distinct complex composition of BAF complexes that interact with SMAD2/3 (Supplementary Figure 4G). SMAD2/3 co-immunoprecipitation experiments indicated an interaction of SMAD2/3 with general subunits SS18 and BRD9 (Figure 2P), and subunits that are specific for embryonic stem cell–specific BAF (esBAF) (BCL11a), neural progenitor BAF (npBAF) (BAF180), and ncBAF (GLTSCR1).

Collectively, these results suggest that SMAD2/3 forms a complex with several of the distinct BAF complexes, which have all BRD9 enzyme as a subunit (Figure 2R).

### Inhibition of BRD9 Suppresses In Vivo PDAC Tumor Formation

To study the effect of BRD9 on PDAC cell growth in vivo, xenograft PDAC mouse models were developed by subcutaneous injection of CSCs from the PDAC line A13A with different transduction/treatment regimens (Supplementary Figure 5A). At 2 months post inoculation, only mice implanted with control adenovirus-transduced cells (containing scrambled control shRNA, A13A^Null^) developed tumors. At this point, no tumors were observed in other mice implanted with gemcitabine-treated cells (A13A^gemcitabine^), shRNA-BRD9 adenovirus-transduced cells (A13A^BRD9-KD^), or A13A^BRD9-KD^ plus with gemcitabine treatment (A13A^BRD9-KD + gemcitabine^), indicating the slower growth rate of these cells in contrast to A13A^Null^. After 3 months of cell inoculation, although no tumors were observed in mice with A13A^BRD9-KD + gemcitabine^, the other mice implanted with A13A^Null^, or A13A^gemcitabine^, or A13A^BRD9-KD^ developed tumors, respectively. To further characterize these tumors, calliper measurement and positron emission tomography–fluorodeoxyglucose F 18 (PET-^18^F-FDG) were performed to analyze the size and metabolic activity of these tumors. As expected, the A13A^Null^ tumors grew much faster than other tumors from month 3 to month 4, as evidenced by the significantly increased tumor volume (Supplementary Figure 5B). Although tumors formed by A13A^gemcitabine^ or A13A^BRD9-KD^ were detected at month 3, they were moderate and grew slowly from month 3 to month 4. Importantly, BRD9-KD further delayed tumor growth compared with A13Al^Null^ and A13A^gemcitabine^ groups. These results were consistent with the observation obtained from PET-^18^F-FDG. At month 4 post inoculation, the ^18^F-FDG uptake in tumors formed by A13A^Null^ was much higher than in other tumors (Supplementary Figure 5C). Gemcitabine significantly decreased the ^18^F-FDG uptake in A13A^gemcitabine^ tumors compared with A13A^Null^ tumors, and BRD9-KD further reduced the ^18^F-FDG uptake of A13A^BRD9-KD^ tumors compared with gemcitabine treatment. Consistently, the tumors harvested from A13A^BRD9-KD^-implanted mice at month 4 were markedly smaller compared with tumors from A13A^Null^ or A13A^gemcitabine^ implanted mice (Supplementary Figure 5D). The lower tumor-weight to body-weight ratios of A13A^BRD9-KD^-transplanted mice further demonstrated a progressive decrease in tumor mass (Supplementary Figure 5E). In contrast, no tumors were detected in any mice with A13A^BRD9-KD + gemcitabine^ and these mice were alive without any signs of sickness at month 4. At necropsy, no metastases were observed among these tumor-bearing mice and non–tumor-bearing mice. Histological examination of these tumor sections by hematoxylin-eosin staining found prominent heterogeneity and extensive necrosis in A13A^Null^ tumors than in A13A^gemcitabine^ tumors, whereas A13A^BRD9-KD^ tumors only showed a small area of necrosis (Supplementary Figure 5F). These data suggested that BRD9 activity drives the tumor progression through regulating PDAC cell growth.

**Figure 3.**
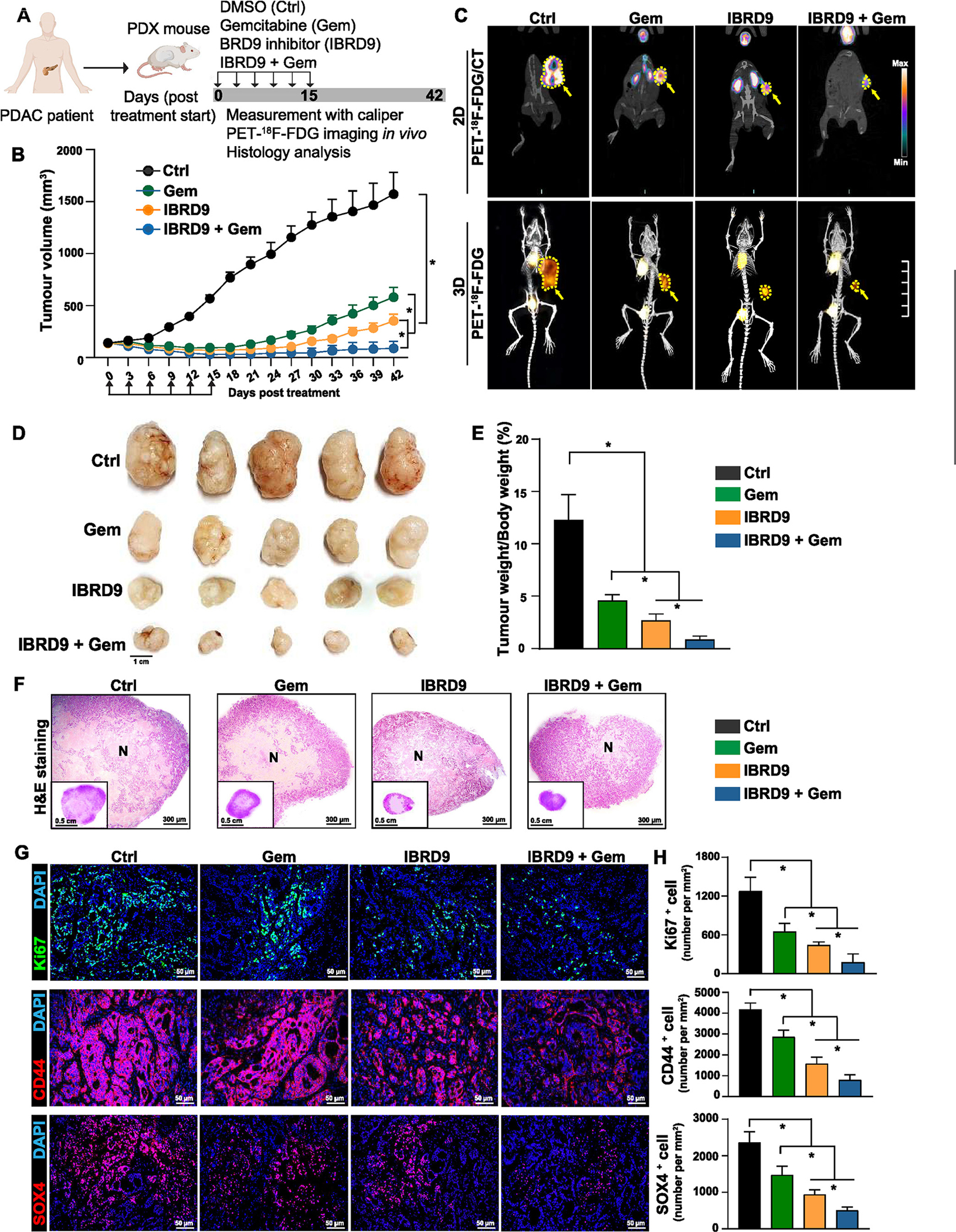
Inhibition of BRD9 enhances antitumor activity in patient-derived xenograft (PDX) models of PDAC. (*A*) Experiment scheme for assessing BRD9 inhibitor in PDX models. (*B*) Tumor growth in PDX models with various treatments (n = 5). Statistical analysis was performed by 2-way analysis of variance (ANOVA) with multiple comparisons with Tukey post-test. *P < .05. (*C*) Representative images of PET-^18^F-FDG /computed tomography scanning. (*D*) Gross morphology of tumors harvested from PDX mouse models. (*E*) Measurement of tumor-weight to body-weight ratio (n = 5). Statistical analysis was performed by 1-way ANOVA with Dunnett’s post-test. *P < .05. (*F*) Representative images of hematoxylin-eosin–stained tumor sections obtained from PDX mouse models. N denotes tumor necrosis. (*G*) Representative images of immunofluorescence staining with Ki67, CD44, and SOX4 antibody in tumor sections obtained from PDX mouse models. (*H*) Quantification data of Ki67, CD44, and SOX4 immunofluorescence staining (n = 5). Statistical analysis was performed by 1-way ANOVA with Dunnett’s post-test. *P < .05.

Although BRD9 inhibition suggested tumor progression through regulating PDAC cell growth in our cell-line xenografts, the cell-line xenografts do not accurately recapitulate the histopathological and molecular characteristics of the human parental tumor. Therefore, human xenograft PDAC models (PDX models) were used to further evaluate the therapeutic potential of BRD9 inhibitor in vivo. Human tumors from a patients with PDAC were implanted into NOD.Cg-*Prkdc*^scid^ *Il2rgtm1Wjl/*SzJ (NOD Scid gamma) host strain. Following transplantation, various treatment schedules were initiated when the tumors reached an average size of 150 mm^3^ with a repeated injection schedule (once every 3 days for a total of 6 treatments within 15 days) (Figure 3A). As expected, gemcitabine (Gem), BRD9 inhibitor (IBRD9), or gemcitabine in combination with BRD9 inhibitor (IBRD9 + Gem) significantly delayed the tumor growth as evidenced by the tumor growth curves and tumor volume (Figure 3B) when compared with DMSO-treated control group (Ctrl). In contrast to the Gem and I-BRD9 group the most striking tumor regressions were observed in the IBRD9 + Gem group. Notably, from day 27 to day 42 after completing treatments, the tumors in the Gem group recurred with rapid growth, whereas most of the xenograft tumors in IBRD9 + Gem group grew slowly over a prolonged period without significant changes in tumor volume (Figure 3B). To monitor the treatment response and tumor progression in living mice, PET-^18^F-FDG was performed (Figure 3C). In the control groups, higher metabolic activity associated with higher ^18^F-FDG uptake was found in these highly proliferative tumors. In contrast, treatment with Gem, IBRD9, or IBRD9 + Gem decreased levels of tumor ^18^F-FDG uptake. In particular, ^18^F-FDG uptake in the IBRD9 + Gem group was significantly lower than in any other group (Figure 3C) indicating that the combination of IBRD9 + Gem significantly delayed the tumor growth. Consistent with reduced tumor growth, the tumor size and tumor-weight to body-weight ratios in IBRD9 + Gem group were significantly lower than those originating from Ctrl and Gem treatment groups (Figure 3D and E). Importantly, IBRD9 treatment or the combination therapy with Gem did not show any toxic effects as no significant alteration of body weight was observed in IBRD9 and IBRD9 + Gem–treated mice (data not shown). Histology analysis of these tumor sections revealed a larger necrosis area in Ctrl and Gem groups as compared with IBRD9 and IBRD9 + Gem groups (Figure 3F), indicating rapid growth of these tumors in Ctrl and Gem groups. In line with these findings, Ki67^+^ proliferating tumor cells were substantially increased in Ctrl and Gem groups, whereas they were reduced in IBRD9 and IBRD9 + Gem–treated tumors (Figure 3G and *H*), suggesting a potential additive effect of BRD9 in controlling primary tumor growth.

We investigated the expression of SOX4 and CD44 in our tumor samples from xenograft mice treated with IBRD9 and in untreated tumors. Immunofluorescence staining of tumor sections indicated a decrease in SOX4 and CD44 expression in tumors of mice treated with IBRD9 compared with untreated tumors. These results confirm the relevance of our ex vivo findings to the in vivo effects (Figure 3G and H).

### BRD9 Inhibition Eliminates CSCs From Patient Tumor

**Figure 4.**
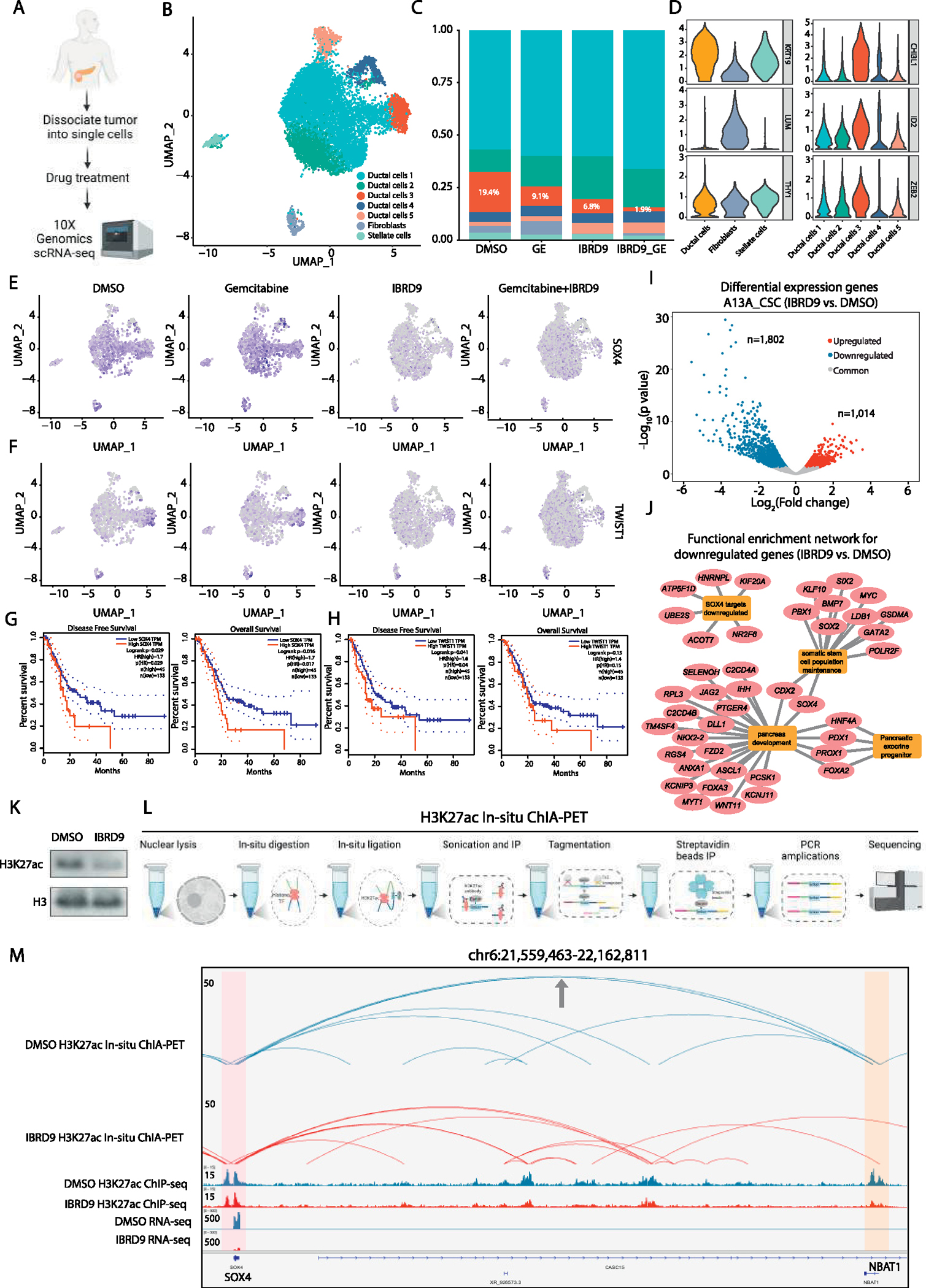
Inhibition of BRD9 reduces CSCs in patient tumors and the enhancer-promoter connectome in CSCs. (*A*) Schematic depiction of single-cell RNA-sequencing strategy. (*B*) Single-cell RNA-sequencing analyses of patient-derived primary PDAC cells with clustering of cells. (*C*) BRD9 inhibition reduces the relative abundance of stem cell–like cells and enhances the effects of gemcitabine. (*D*) Violin plots corresponding to marker gene expression in the different cell types in clusters. (*E* and *F*) BRD9 inhibition leads to the elimination of cells that express CSC genes *(E*) *SOX4* and (*F*) *TWIST1*. (*G* and *H*) Higher expression of *SOX4* and *TWIST1* correlate with lower survival of patients with pancreatic cancer, and lower disease-free survival based on The Cancer Genome Atlas data. (*I*) Differential gene expression analysis on BRD9 inhibition in CSCs. (*J*) Functional enrichment network of downregulated genes on BRD9 inhibition. (*K*) BRD9 inhibition reduces the abundance of H3K27ac in CSCs. (*L*) Schematic depiction of analyzing enhancer-promoter connectivity by H3K27ac in situ chromatin interaction analysis with paired-end tag sequencing, H3K27ac chromatin immunoprecipitation sequencing (ChIP-seq) and RNA-sequencing (RNA-seq). (*M*) Genome browser view of H3K27ac loops *for SOX4* genes*. SOX4* promoter is highlighted in pink and *NBAT1* promoter (enhancer-like) is highlighted in orange.

To further examine the translational relevance of BRD9 as a candidate therapeutic target, we used freshly isolated primary cancer patient samples and treated them with I-BRD9 or in combination with gemcitabine for 72 hours followed by single-cell RNA-sequencing (Figure 4A) for ~12,000 cells. The analysis of this single-cell RNA-sequencing data indicated different populations of cells based on their expression of genes characteristic for ductal cells (*KRT19*), fibroblasts (*LUM*), and stellate cells (*THY1*), and the further separation of pancreatic ductal cells to different subpopulations of cancer cells (Figure 4B and D). One of these ductal cell subpopulations, designated as ductal cells 3, expressed a range of CSC markers. This cancer cell subpopulation was reduced by I-BRD9 treatment (from 19.4% to 6.8%) and showed a 10-fold reduction in numbers (from 19.4% to 1.9%) on cotreatment with I-BRD9 and gemcitabine (Figure 4C). Enriched Gene Ontology terms for downregulated genes in conditions I-BRD9/gemcitabine and I-BRD9 vs DMSO and gemcitabine indicated central mechanisms that are relevant for PDAC development and CSCs. These terms included extracellular matrix organization, cell proliferation, cell migration, cell adhesion, regulation of apoptotic signaling pathways, and regulation of angiogenesis (Supplementary Figure 6A), all of particular relevance for tumor aggressiveness and metastatic processes. The enriched pathways for downregulated genes in these same conditions uncovered TGF*β*/Activin A signaling pathway, fibrosis, vascular endothelial growth factor A signaling, wound healing, autophagy pathways in cancer, proinflammatory/profibrotic mediators, nuclear factor-*k*B, and YAP1/ECM axis (Supplementary Figure 6B). Among the genes that are specifically repressed by I-BRD9 treatment in the CSC population of cells were *SOX4* and *TWIST1* (Figure 4E), and *SNAIL2*, *JUN*, *IL6*, and *IGF1* (Supplementary Figure 6C). The combined treatment of I-BRD9 and gemcitabine resulted in a synergistic reduction of these CSC factors (Figure 4E and Supplementary Figure 6C). Quantitative polymerase chain reaction (qPCR) analyses indicated a reduction of these markers in all 6 cell lines on I-BRD9 treatment, thus providing further confirmation of the effects of I-BRD9 on CSC gene expression (Supplementary Figure 6E). In addition, we found a number of factors that have been described as regulators of CSC self-renewal factors.^47^ These include *HOXA4, HOXA5, HOX3A, YAP1, MSI2, HIF1A, NOTCH2, MEIS1, OCT4,* and *NES,* which are known as self-renewal factors in CSCs in various cancers, and enriched by gemcitabine treatment but decreased by I-BRD9 treatment, particularly on the combined treatment with gemcitabine and I-BRD9 (Supplementary Figure 7A). Besides self-renewal factors, we found the enrichment of EMT-inducing transcription factors *SNAI1, SNAI2, TWIST2, ZEB1,* and *ZEB2* that promote metastatic dissemination of CSCs (Supplementary Figure 7B).

We also compared the transcriptional features of different tumor subsets identified from our single-cell RNA-sequencing experiments to those identified from other studies.^48^ We found that the populations of cells changing under IBRD9 treatment overlap with gene expression signatures of the basal subtype of PDACs (sig.2) and an EMT signature (Supplementary Figure 7C), which has been found to correlate with higher mortality, chemotherapy resistance, and higher metastatic capacity. These data indicate that the CSC population eliminated by I-BRD9 in our data are more reminiscent of basal subtype of cells overlap with the aggressive subtype of PDACs described by Chan-Seng-Yue et al^48^ and other studies.

Altogether, the results using resected patient tumor samples indicated that BRD9 inhibition by a small molecule compound can efficiently target and eliminate the CSC subpopulation of pancreatic cancer cells, thus confirming our prior discoveries on PDAC cell lines.

### BRD9 Inhibition Disrupts Enhancer-Promoter Connectome of Stemness-related Genes in CSCs

Next, we performed bulk RNA-sequencing on I-BRD9 inhibition in A13A CSCs which identified 1802 downregulated genes and 1014 upregulated genes (Figure 4I). The functional enrichment network analysis of the downregulated genes by BRD9 inhibition revealed a number of central transcriptional regulators of somatic stem cell maintenance (eg, *SOX4, SOX2, MYC, BMP7*), pancreatic exocrine progenitors/pancreatic development (eg, *DLL1, JAG2, WNT11, NKX2–2*), and SOX4 target genes (Figure 4J). Gene set enrichment pathway analysis of I-BRD9 downregulated genes indicated SMAD3-mediated transcription and EMT (Supplementary Figure 8A) as enriched pathways. Altogether, these results emphasize the importance of key processes involved in regulating stem cell maintenance and metastasis that are downregulated on BRD9 inhibition.

**Figure 5.**
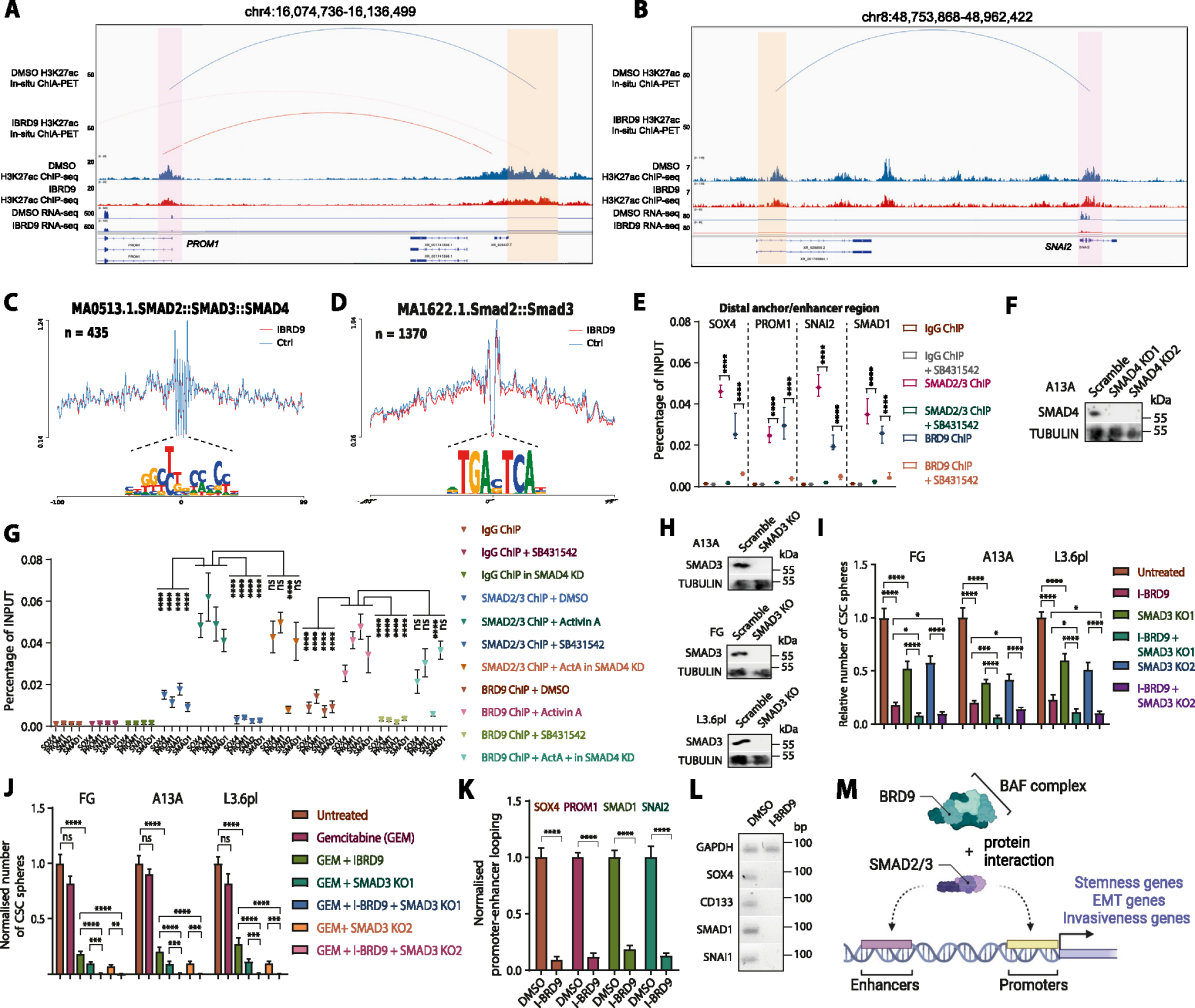
BRD9 regulates enhancer-promoter connectome of stemness and EMT loci in CSC via cooperating with SMAD2/3. (*A* and *B*) BRD9 inhibition leads to the loss of enhancer-promoter looping and loss of expression at stem cell and EMT loci such as CD133/PROM1, SMAD1, and SNAI2. The genomic loci show H3K27ac abundance and gene transcription together with 3D chromatin interactions. (*C* and *D*) Transcription factor footprint analysis identified SMAD3 footprints based on JASPAR motif database at the ATAC-seq peaks of the anchor regions. (*C*) MA0513 has 254 hits in Ctrl-specific ATAC peaks and 211 hits in I-BRD9-specific ATAC peaks on anchors. (*D*) MA1622 has a total of 1370 hits in control-specific ATAC and I-BRD9-specific ATAC peaks on anchors. The depth of the footprinting is not statistically different in control and BRD9-I treated samples. (*E*) TGF*β*/Activin signaling leads to the loss of SMAD2/3 and BRD9 binding to the 3D chromatin looping anchors at enhancers of stem cell and EMT loci in CSCs. Chromatin immunoprecipitation (ChIP)-qPCR of distal anchor regions at SOX4, CD133/PROM1, SNAI2, and SMAD1 loci in CSCs ± SB431542. (*F*) SMAD4 knockdown clones in A13A cell line. (*G*) The effect of SMAD4 knockdown and TGF*β*/Activin signaling on SMAD2/3 and BRD9 binding to target loci. (*H*) SMAD3 knockout clones in 3 PDAC cell lines. (*I*) The crosstalk between of BRD9 inhibition and SMAD3 knockout on CSC self-renewal. (*J*) The effect of SMAD3 knockout on BRD9 inhibition on chemoresistance. (*K* and *L*) 3C-qPCR analysis and gel electrophoresis of qPCR product of enhancer-promoter looping on *SOX4, CD133, SMAD1 SNAI1,* and loci on BRD9 inhibition. GAPDH locus was used as an internal control. Statistical analysis was performed by 2-way analysis of variance with multiple comparisons with Tukey correction. ****Adjusted *P* < .0001, ***adjusted *P* < .001, **adjusted *P* < .01, *adjusted *P* < .05. (*M*) Schematic depiction of the function of SMAD2/3 and BRD9 in regulating the chromatin looping between enhancer and promoter regions near stemness genes, EMT, and invasiveness genes.

In a cellular context, transcriptional output is largely orchestrated by *cis*-regulatory elements (CREs), in particular enhancers and promoters. By performing Assay for Transposase-Accessible Chromatin using sequencing (ATAC-seq), we identified that I-BRD9 treatment in A13A CSCs results in extensive loss of CREs (n = 1609) compared with control samples (Supplementary Figure 6D). In line with this, Western blots demonstrated that acetylated lysine27 at histone 3 (H3K27ac),^49^ an important active enhancer and promoter histone mark, was significantly decreased in response to BRD9 inhibition (Figure 4K). In most cases, enhancers control gene expression through long-range interactions with promoters,^50,51^ but very little is known about the enhancer/promoter connectome in pancreatic CSCs. To study the effects of BRD9 inhibition on the enhancer/promoter connectome of CSCs, we performed H3K27ac in situ chromatin interaction *analysis* with paired-end tag sequencing^52^ experiments, which capture H3K27accentric chromatin interactions (ie, enhancer/promoter connectome) (Figure 4L). These data suggest that the transcriptional downregulation of stemness-related genes is possibly due to the loss of long-range enhancer-promoter connectome. For example, we found that in control CSCs, the *NBAT1* promoter is characterized by H3K27ac enrichment on the promoter but is transcriptionally silenced. In addition, the *NBAT1* promoter has strong chromatin interaction with the *SOX4* promoter, suggesting that this non-transcribing promoter may function as an enhancer to regulate the expression of SOX4. However, I-BRD9 treatment diminished the H3K27ac enrichment on the *NBAT1* promoter and its physical contact with the *SOX4* promoter, which possibly contributes to the transcriptional downregulation of *SOX4* (Figure 4M). Similarly, the transcriptional downregulation of other stemness-related genes (eg, *CD133/PROM1, SMAD1,* and *SNAI2*) is linked to the loss of enhancer-promoter connectome (Figure 5A and B; Supplementary Figure 8B and C).

ATAC-seq data showed SMAD2/3 footprints at the anchor regions (Figure 5C and D). SMAD2/3 footprints, SMAD2/3 phosphorylation, SBE4-luc activity, or TGF*β* target genes SMAD7, SERPINE1, SERPINE2, and SKIL were not affected by BRD9 inhibition (Supplementary Figure 8D–G). In contrast, IBRD9 reduced the expression of SNAI2, SNAI1, and TWIST2, suggesting a particular impact on certain genes such as the EMT regulatory circuitry. BRD9 and SMAD2/3 bound to the anchor regions, whereas the binding of BRD9 on the anchor regions was lost following TGF*β*/Activin pathway inhibition. In turn, Activin A treatment further increases SMAD2/3 and BRD9 binding to *SOX4, PROM1, SNAI2,* and *SMAD1* loci, whereas the binding is blocked on the inhibition of TGF*β* signaling pathway with SB431542 (Figure 5E). Our findings revealed that the binding of SMAD2/3 on the SNAI2 locus was decreased in A13A SMAD4 knockdown cells, whereas there were no significant differences in binding on the *SOX4, PROM1,* and *SMAD1* loci (Figure 5F and G). Overall, these findings suggest that SMAD4 deletion can affect a subset of SMAD2/3-BAF targeted loci, potentially contributing to clonal evolution and diversification of PDACs.

To clarify whether the BRD9 and SMAD2/3 interaction was indispensable for the role of BRD9 in CSCs, we performed SMAD3 knockout in 3 PDAC cell lines (Figure 5H), and studied its impact on CSC self-renewal and chemoresistance. The knockout of SMAD3 led to a moderate reduction in CSC self-renewal but a strong reduction in chemoresistance to gemcitabine (Figure 5I and *J*). Furthermore, the combined SMAD3 knockout with BRD9 inhibition further reduced the self-renewal and particularly the chemosensitivity.

Once recruited to the regulatory regions, the BRD9/BAF complex might facilitate the 3D chromatin looping of enhancers and promoters, and the stable binding of additional epigenetic regulatory complexes that deposit H3K4me3, H3K27ac, and H3K36me3 onto stemness loci. In support of this, chromatin immunoprecipitation qPCR of DPY30 of the TrxG complex and CREB-binding protein indicates that BRD9 inhibition and SMAD2/3 pathway inhibition reduces TrxG and CREB-binding protein presence on BRD9 target loci such as *SOX4* (Supplementary Figure 8H), whereas 3C experiments on *SOX4, PROM1, SNAI2,* and *SMAD1* loci indicate a decrease in promoter-enhancer looping on BRD9 inhibition (Figure 5K and L).

These results indicate that BRD9 and SMAD2/3 are co-binding to these regions and SMAD2/3 transcription factors recruit BRD9 to the gene promoter and enhancer regions (Figure 5M). Collectively, our results revealed the cooperation of BRD9 and SMAD2/3 in regulating the enhancer-promoter connectome and gene expression of stemness-related genes in pancreatic CSCs.

## Discussion

Differentiation and dedifferentiation processes mediated by transcription factors and epigenetic regulatory proteins can contribute to tumor formation, invasiveness, metastatic processes, dormancy, and reactivation of cancer cells; clonal evolution of tumor cells; and the development of therapeutic resistance in cancers.^4^ Of particular importance to all these processes, epigenetic mechanisms regulate phenotypic plasticity and the self-renewal capacity of CSCs. Accordingly, targeting epigenetic mechanisms offers an attractive strategy to eliminate CSCs through reducing their self-renewal characteristics or resensitizing them for chemotherapeutic drugs.

We used a compound screening approach to identify candidate targets that regulate stem cell–like characteristics of CSCs. Using this strategy, we identified BRD9 as a novel epigenetic regulator of CSC and the corresponding small molecule inhibitors for this enzyme. Our experiments uncovered that BRD9 as a subunit of ncBAF, esBAF, and npBAF cooperates with TGF*β*/Activin-SMAD2/3 in regulating CSC self-renewal, chemoresistance, and invasiveness. SMAD2/3 recruits the BAF complexes to the expression of stem cell loci by regulating the 3D enhancer and promoter interactions in CSCs. esBAF functions in regulating the self-renewal and pluripotency of embryonic stem cells, whereas npBAF is important for neural progenitor/neural stem cells, and noncanonical BAF is linked to naïve pluripotent stem cells.^46^ This could reflect the developmentally plastic state of CSCs and their capacity to reversibly differentiate and dedifferentiate by using BAF complexes. The dynamics of different SMAD2/3-BAF complexes could provide the necessary stem cell–like developmentally plastic or “metastable” capacity of CSCs, as we have previously shown in human embryonic stem cells for mixed lineage leukemia/COMPASS.^24^ Because BAF subunits ARID1A and BAF180/Polybromo are mutated in a subset of PDACs, it suggests possibly divergent mechanisms of how the BAF complexes could promote CSC characteristics in PDACs. Furthermore, SMAD4 as an important cofactor of SMAD2/3 and component of the TGF*β*/Activin signaling pathway is mutated in approximately half of PDACs.^22^ This could indicate differences in the molecular mechanisms of how CSCs are epigenetically regulated on SMAD4 mutation.

An interesting characteristic of tissue-specific stem cells is their capacity to enter and exit a quiescent stage. This inability to slow down or exit the proliferative cycle in G1 and enter the quiescent state G0, makes the cells susceptible for gemcitabine-mediated killing. Interestingly, the G0 phase in the PDAC CSCs has only a low p27(K−) induction. This could reflect that cells are temporarily in a “shallow quiescent” state, which has been termed “GAlert” or “primed” cells. Such cells can reenter G1 and the cell cycle faster than more “deeply quiescent” cells.^43^

BRD9 inhibition could be used in combination with currently available treatments such as gemcitabine or FOLFIRINOX with the objective of resensitizing pancreatic CSCs that are particularly resistant to currently used chemotherapies. Hence, the improved molecular target identification serves a dual purpose; in addition to improving drug selectivity, our discoveries will guide effective therapy combination with either standard of care or other investigational agents. The determination of the CSC 3D chromatin architecture provides insights into higher levels of epigenetic regulation of gene expression. Our results indicated the SMAD2/3-BAF complex regulates promoter-enhancer connections in CSCs. Therefore, targeting the regulators of enhancer-promoter connectomes is thus emerging as an attractive therapeutic strategy for eliminating the stem cell–like populations in PDACs. Our findings can potentially translate into clinical benefit for PDAC patients who have a surgically unresectable cancer and even undergoing relapse.

## Supplementary Material

Supplemental-2

## Data Availability

The datasets generated during the current study are publicly available on GEO (Accession number: GSE222952). Other analytic methods, including statistical codes, software information, and algorithms used in this study, will be made available on reasonable request to the corresponding author. Further information and requests for resources and reagents should be directed to and will be fulfilled by the lead contact, Siim Pauklin (siim.pauklin@ndorms.ox.ac.uk).

## WHATYOUNEEDTOKNOW

### BACKGROUND AND CONTEXT

Pancreatic ductal adenocarcinoma has a poor prognosis due to the presence of pancreatic cancer stem cells that are resistant to current chemotherapy treatments. The epigenetic mechanisms controlling cancer stem cells are not well understood, hindering the development of effective strategies to eliminate cancer stem cells.

### NEW FINDINGS

Through small molecule compound screening, the bromodomain-containing protein BRD9 was identified as a key chromatin regulator in pancreatic cancer stem cells. BRD9 works in conjunction with the transforming growth factor *β*/Activin-SMAD2/3 signaling pathway to maintain the stemness of cancer stem cells. Inhibition or genetic ablation of BRD9 prevents cancer stem cell self-renewal, cell cycle entry into G0 phase, and invasiveness, while also increasing sensitivity to gemcitabine treatment. Pharmacological inhibition of BRD9 reduces tumorigenesis in mouse models and eliminates cancer stem cells in tumors from patients with pancreatic cancer. The inhibition of BRD9 disrupts enhancer-promoter looping and transcription of stemness genes in cancer stem cells.

### LIMITATIONS

While the study provides promising results regarding BRD9 as a therapeutic target, further research is needed to fully understand its clinical implications and potential side effects.

### CLINICAL RESEARCH RELEVANCE

The identification of BRD9 as a key regulator of cancer stem cell stemness in pancreatic ductal adenocarcinoma opens up new possibilities for targeted therapies that can eliminate cancer stem cells and improve treatment outcomes for patients.

### BASIC RESEARCH RELEVANCE

This study contributes to a better understanding of the epigenetic mechanisms involved in cancer stem cell regulation, specifically in the context of pancreatic cancer.

## Abbreviations used in this paper:

3D: 3-dimensional
ATAC-seq: Assay for Transposase-Accessible Chromatin using sequencing
CSC: cancer stem cell
dBRD9: degrader of BRD9
DMSO: dimethyl sulfoxide
EMT: epithelial-to-mesenchymal transition
esBAF: embryonic stem cell–specific BAF
KD: knockdown
ncBAF: noncanonical BAF chromatin remodeling complex
npBAF: neural progenitor BAF
PDAC: pancreatic ductal adenocarcinoma
PET-^18^F-FDG: positron emission tomography–fluorodeoxyglucose F 18
qPCR: uantitative polymerase chain reaction
shRNA: short hairpin RNA
TGF: transforming growth factor
