# Supplementary material for: BRD9-SMAD2/3 Orchestrates Stemness and Tumorigenesis in Pancreatic Ductal Adenocarcinoma
Source: Gastroenterology. Author manuscript; Available in PMC 2024 Aug 7. (PMC11304550; doi:10.1053/j.gastro.2023.09.021)
Supplement: Supplemental-2 [file NIHMS2008921-supplement-Supplemental-2.pdf]

Supplementary Materials and Methods

Cell lines and cell culture

The A13A, A13D, A13B cells were provided by Christine Iacobuzio-Donahue at The Memorial Sloan Kettering Cancer Center. FG and L3.6 were provided by Isaiah J. Fidler at The University of Texas MD Anderson Cancer Center. For standard cell cultures, cells were grown at 37°C humidified incubator containing 5% CO2 in Dulbeccos modified Eagles medium (DMEM), high glucose, GlutaMAX™ Supplement, pyruvate (Thermo Fisher Scientific, Inc.), 100 U/ml penicillin, 100 mg/ml streptomycin (Thermo Fisher Scientific, Inc.), MEM Non-Essential Amino Acids Solution 1X (Life Technologies; Thermo Fisher Scientific), MEM Vitamin Solution 1X (Life Technologies; Thermo Fisher Scientific) and 10% inactivated fetal calf serum (FCS; Life Technologies). For 3D cultures, cells we grown in ultra-low attachment plates (Thermo Fisher Scientific, Inc.) in Dulbecco's Modified Eagle Medium/F12 (Sigma-Aldrich) supplemented with 2.5mM L-Glutamine (Thermo Fisher Scientific, Inc.), 1 % B27 supplement (Life Technologies), 100 U/ml penicillin, 100 mg/ml streptomycin (Thermo Fisher Scientific, Inc.), 20ng/ml basic thermostable fibroblast growth factor (FGF-Basic TS, Proteintech) (5,000 cells/ml).

Tumour sphere assays

Cells are seeded into ultra-low attachment plates in Dulbecco's Modified Eagle Medium/F12 (Sigma-Aldrich) supplemented with 2.5mM L-Glutamine (Thermo Fisher Scientific, Inc.), 1 % B27 supplement (Life Technologies), 100 U/ml penicillin, 100 mg/ml streptomycin (Thermo Fisher Scientific, Inc.), 20ng/ml basic thermostable fibroblast growth factor (FGF-Basic TS, Proteintech) (5,000 cells/ml). For tumour sphere formation assay, cells were passaged after one-week incubation, and grown for another week after which the tumour sphere numbers were counted under a phase-contrast microscope using the 40x magnification lens or by Celigo Image Cytometer (Nexcelom). For RNA-seq and ATAC-seq experiments, serial passaging of the first-generation tumour spheres is required. After 7 days of incubation the tumour spheres were harvested by using a 40 µm cell strainer and centrifuged for 5 min at 200 x g at RT. Dissociate the pellet of tumour spheres to single cells using trypsin, and then expanded for another 4 days before performing the treatments of samples.

Knockin cell lines for Oct4

pCCC construct and OCT4 TALEN constructs (pTALEN_V2-OCT4F, pTALEN_V2-OCT4R) constructs were a gift from Francis C. Lynn and have been published [1]. OCT4-eGFP-PGK-Puro was

a gift from Rudolf Jaenisch (Addgene plasmid #31937) and have been published [2]. Cells were transfected with Lipofectamine 3000 (ThermoFischer Scientific) and cultured for 4 days after transfection before selecting with 0.25 µg/mL puromycin (Sigma). Colonies were individually picked, trypsinized and placed into 24-well plates with 500 µl of media. Once clones were close to confluent, cells were replica plated for genotyping, freezing and for expanding the correctly targeted clones. Genomic DNA was extracted using Promega Wizard SV Genomic DNA Purification System (Promega) and genotyping was performed as described [1]. Positive clones were analysed by flow cytometry to estimate the frequency of eGFP positive cells in the cancer cell population. We additionally used primers GGTGCTCAGGTAGTGGTTGTCG and CTCTAATGTCCTCCTCTAACTGCTCTAGG for Oct4-GFP region verification, as well as CACAACCTCCCCTTCTACGAGC and GCATCATTGAACTTCACCTTCCCTC for Oct4-Puromycin region verification.

The small molecule screening library

The screening was performed on FG cells with an in-frame eGFP knock-in into the in the endogenous OCT4 locus resulting in an OCT4-eGFP fusion protein upon expression. The screening library contained concentrated small molecule compounds with verified biochemical activity against their targets. Most of the compounds target epigenetic regulators with high specificity (Supplementary Table 1).

Supplementary Table 1. Small molecule compounds used in the screening experiment. List with compound names, working concentrations and target molecules.

Vial	Source Name	[Working] uM	Class/Target
1	(+)-JQ1	1	Bromodomains - BRD2, BRD3, BRD4, BRDT (BET)
2	(-)-JQ1 (inactive)	1	Bromodomains - Negative control
3	PFI-1	5	Bromodomains - BRD2, BRD3, BRD4, BRDT (BET)
4	I-BET	1	Bromodomains - BRD2/3/4
5	Bromosporine	1	Bromodomains - pan-Bromodomain
6	CBP/BRD4 (0383)	5	Bromodomains - CBP, BRD4(1)
9	SGC-CBP30	1	Bromodomains - CREBBP, EP300
10	I-CBP112	1	Bromodomains - CREBBP, EP300
15	RVX-208	5	Bromodomains - BRD2, BRD3, BRD4, BRDT (BET, BD2)
16	SMARCA	2.5	Bromodomains - SMARCA, PB1
17	PB1/SMARCA	1	Bromodomains - SMARCA, PB1
18	PFI-3	1	Bromodomains - SMARCA2/4, PB1(5)
19	GSK2801	1	Bromodomains - BAZ2A, BAZ2B
20	PFI-4	1	Bromodomains - BRPF1B

21	TRIM24/BRPF	10	Bromodomains - TRIM24/BRPF
22	OF-1	5	Bromodomains - pan-BRPF
23	Belinostat	5	HDAC - hydroxamic acids
24	CXD101	1	HDAC -
25	Valproic acid	1000	HDAC - aliphatic acid compounds
26	Entinostat	0.5	HDAC - ortho-amino anilides
27	SAHA	2.5	HDAC - hydroxamic acids
28	Trichostatin A	0.5	HDAC - hydroxamic acids - Class I & II
29	SRT1720	1	HDAC - SIRT1 (indirect?) activator
30	EX 527	1	HDAC - SIRT1
32	CI-994	1	HDAC - 1,2,3,(8)
33	CPI-360	10	Histone methyltransferase - EZH2 and EZH1
34	CPI-413	10	Histone methyltransferase - EZH2 and EZH1
35	UNC0638	1	Histone methyltransferase - G9a, GLP
37	UNC0642	1	Histone methyltransferase - G9a, GLP
38	A-366	2	Histone methyltransferase - G9a, GLP
39	Chaetocin	0.05	Histone methyltransferase - SUV39H1
43	PFI-2	2	Histone methyltransferase - SETD7
45	SGC0946	7.5	Histone methyltransferase - DOT1L
46	GSK343	3	Histone methyltransferase - EZH2
47	UNC1999	1	Histone methyltransferase - EZH2
49	LLY-507	1	Histone methyltransferase - SMYD2
50	Tranylcypromine	20	Lysine demethylases - LSD1
51	GSK-LSD1 (irreversible)	0.5	Lysine demethylases - LSD1
52	GSK690	5	Lysine demethylases - LSD1
53	GSK J4	10	Lysine demethylases - JMJD3, UTX, JARID1B
54	GSK J5 (inactive)	10	Lysine demethylases - Negative control
55	IOX1 (5-carboxy-8HQ)	40	Lysine demethylases - pan-2-OG
57	Methylstat (Ester)	2.5	Histone demethylase
58	(E)-JIB-04	0.05	Histone demethylase - Pan JmjC
60	ML324	5	Histone demethylase - JMJD2E
61	IOX2	10	Prolyl-Hydroxylases - PHD2 (EGLN1)
62	OICR-9429	1	Methyl Lysine Binder? - WDR5
63	UNC1215	5	Methyl Lysine Binder - L3MBTL3
64	5-Azacitidine	10	DNA methyltransferase (DNMT) -
65	5-Azadeoxycitidine	5	DNA methyltransferase (DNMT) - DNMT1/3
66	Olaparib	1	Poly ADP ribose polymerase (PARP)
67	Rucaparib	10	Poly ADP ribose polymerase (PARP)
68	K00135	1	Kinase inhibitor - ATP competitive - PIM
69	5-Iodotubercidin	1	Kinase inhibitor - ATP mimetic - Haspin
70	C646	1	Histone acetyltransferase (HAT) p300/CBP
74	DUAL1946	1	
75	GSK484	1	Peptidyl arginine deiminase (PAD4)
76	KDOBA67	10	Histone demethylase
77	BAZ2-ICR	1	Bromodomains - BAZ2A, BAZ2B
78	NI-57	1	Bromodomains - pan-BRPF

79	LP99	1	Bromodomains - BRD9, BRD7
80	SGC707	1	Arginine methyltransferase - PRMT3
81	RGFP966	10	HDAC - HDAC3
82	PCI-34051	5	HDAC - HDAC8
83	Rocilinostat	10	HDAC - HDAC6
84	Tubastatin A HCl	10	HDAC - HDAC6
85	KDOAM-25a	1	Lysine demethylases - JARID
86	KDM5-C70	10	Histone demethylase - JARID1
88	MAZ1805	1	t-RNA sythetase
89	MAZ1392	1	t-RNA sythetase
90	BI-9564	1	Bromodomains - BRD9, BRD7
91	NVS-CECR2-1	1	Bromodomains - CECR2
92	GSK106	1	Peptidyl arginine deiminase (PAD4)
93	J556-42R	1	Arginine methyltransferase - PRMT5
94	J556-63R	1	Arginine methyltransferase - PRMT5
95	J556-70R	1	Arginine methyltransferase - PRMT5
96	A-196	1	Histone methyltransferase - SUV420H1/H2
97	BAY-598	1	Histone methyltransferase - SMYD2
98	J556-143	1	Arginine methyltransferase - PRMT5
99	MS049	1	Arginine methyltransferase
100	MS023	1	Arginine methyltransferase - Type I PRMTs
101	MS003	1	Arginine methyltransferase - negative control
104	SGI-1776	10	Kinase inhibitor - Haspin
105	CHR-6494	1	Kinase inhibitor - Haspin
106	CPI-169	10	Histone methyltransferase - EZH2, EZH1
107	UNC2400	1	Histone methyltransferase - EZH2
108	GSK864	5	Dehydrogenase
109	GSK8814	10	Bromodomains - ATAD2
110	GSK8815	10	Bromodomains - ATAD2
111	GSK959	1	Bromodomains - BRPF1
112	NVS-CECR2-C	1	Bromodomains - CECR2
113	BAY-299	1	Bromodomains - BRD1, TAF1
114	PCI-24781	10	HDAC -
115	Romidepsin	1	HDAC -
116	Mocetinostat	10	HDAC -
117	Santacruzamate	50	HDAC 2?
118	KDOAM32	1	Lysine demethylases - JARID
120	MS409N	1	Arginine methyltransferase - PRMT4, PRMT6 inactive control
121	TP-064	1	Arginine methyltransferase - PRMT4
122	TP-064N	1	Arginine methyltransferase - PRMT4
123	A-395	1	Methyl Lysine Binder - EED
124	A-395N	1	Methyl Lysine Binder - EED
125	I-BRD9	10	Bromodomains - BRD9
126	TP-472	1	Bromodomains - BRD9
127	TP-472N	1	Bromodomains - BRD9
128	KDOPZ-32a	1	Lysine demethylases - KDM5

129	KDOOA012000	1	Lysine demethylases KDM2
130	AMI-1	50	Arginine methyltransferase - PRMT
131	TMP269	10	HDAC -4, 5, 7 &9
132	AGK2	10	HDAC - SIRT2
133	GSK6853	1	Bromodomains - BRPF1/2/3
134	GSK9311	1	Bromodomains - BRPF1/2/3
135	LLY-283	1	Arginine methyltransferase - PRMT5
136	TD001851a	1	Methyl Lysine Binder/tudor domain -Spin1
137	TDOSI000058a	1	Methyl Lysine Binder/tudor domain -Spin1
138	TD001863a	1	Methyl Lysine Binder/tudor domain -Spin1
139	TDOSI000062a	1	Methyl Lysine Binder/tudor domain -Spin1
140	TD001857a	1	Methyl Lysine Binder/tudor domain -Spin1
141	TD001856a	1	Methyl Lysine Binder/tudor domain -Spin1
142	TD001858a	1	Methyl Lysine Binder/tudor domain -Spin1
143	TMP195	1	HDAC -4,5,7,9
144	GSK2879552	10	Lysine demethylases - LSD1
145	TDO20821a	1	Methyl Lysine Binder/tudor domain -Spin1
146	TDO20824a	1	Methyl Lysine Binder/tudor domain -Spin1
147	TDO20823a	1	Methyl Lysine Binder/tudor domain -Spin1
148	A-485	1	Histone acetyltransferase (HAT) p300/CBP
149	A-486	1	Histone acetyltransferase (HAT) p300/CBP
150	GSK4027	1	Bromodomains - PCAF, GCN5
151	GSK4028	1	Bromodomains - PCAF, GCN5
152	L-Moses	1	Bromodomains - PCAF, GCN5
153	D-Moses	1	Bromodomains - PCAF, GCN5
154	PFI-5	1	Histone methyltransferase - SMYD2
155	YX39-31b	1	Methyl Lysine Binder/tudor domain -Spin1
156	TDO208229	1	Methyl Lysine Binder/tudor domain -Spin1
157	TDO01856a	1	Methyl Lysine Binder/tudor domain -Spin1
158	TDO20826a	1	Methyl Lysine Binder/tudor domain -Spin1
159	Bortezomib	0.1	Protesome
160	Carfilzomib	0.1	Protesome
161	RTS-V5	1	Protesome and HDAC
162	dBRD9	1	Bromodomains - BRD9
163	BI-7273	0.1	Bromodomains - BRD9/7 (IC50 19/117nM)
164	CPI-621	0.1	Lysine demethylases - KDM5

Screening of the chemical compounds

The cells were grown in 96-well plates in standard growth medium with puromycin (1 µg/ml stock). Three technical replicates and three biological replicates were used for the screening. Cells were plated at a concentration of 10,000 cells in 100 µl of media per well in a 96-well plate. One day after

plating the cells, the medium was changed to 90 µl standard growth medium supplemented with puromycin (0.5 µg/ml) and Activin A (10 ng/ml). On the same day, the compounds were added: first, 100x compound library dilutions were made, and 10 µL of 100x diluted chemical was added to each well to obtain 1000x final dilution of the compounds. Cells were then cultured with chemical compounds for five days with media change at day 0, day 2 and day 4 supplemented by fresh compounds. Each replicate was analyzed using Celigo Image Cytometer (Nexcelom) and flow cytometry. Cells were lifted and dissociated into single cells with Trypsin. Details on the antibodies that were used for flow cytometry are listed in Supplementary Table 2. The cells were incubated with 0.5 ug /ml final concentration of conjugated antibodies in 1% BSA-PBS for 40 minutes on ice and washing was repeated as before. The cells were then suspended in 300 uL 1% BSA-PBS with DAPI (1:2000) for live/dead separation and kept on ice to be used for the flow cytometry analysis.

Supplementary Table 2. Antibodies used for the detection of GFP-OCT4, CD133 and SSEA4 by flow cytometry in the compound screening experiment.

Antibody	Target/function	Ratio	Number of cells
CD133-BV786, Mouse Anti-Human, clone W6B3C1, BD 747640	CD133	0.6 µL in 100 µL	300,000
Mouse IgG1-BV786l, BD 563330	isotype control for CD133	0.6 µL in 100 µL	300,000
Alexa647 Mouse anti-SSEA-4 clone MC813-70	SSEA4	2.5 µL to 100 µL	300,000
Alexa647 Mouse IgG3	isotype control for SSEA4	0.31 µL to 100 µL	300,000

Gene knockdown

The stable knockdown of BRD9 was performed by commercial shRNA constructs (Merck): TRCN0000236473; TRCN0000131081; TRCN0000127634. shRNA plasmid DNA was transfected into cells with Lipofectamine 3000 (Thermo Fischer Scientific) according to manufacturer guidelines. Puromycin was added to the growth media at 0.1ug/ml concentration and individual colonies were picked, expanded, and screened for gene knockdown compared to Scramble control transfected cells. The primers used for qPCR verification for BRD9 were as follows: Forward: CGCAGGCTTTAAGATGATGAGC, reverse: GCTCCTCTGCGGTACTGTC.

DNA constructs

pRetroSuper Smad4 was a gift from Joan Massague (Addgene plasmid # 15727; http://n2t.net/addgene:15727; RRID:Addgene_15727). RetroSuper shSmad4J hygro was a gift from Joan Massague (Addgene plasmid # 19151; http://n2t.net/addgene:19151 ; RRID:Addgene_19151). SBE4-Luc was a gift from Bert Vogelstein (Addgene plasmid# 16495; http://n2t.net/addgene:16495; RRID:Addgene_16495). gRNA Cloning Vector was a gift from George Church (Addgene plasmid# 41824; http://n2t.net/addgene:41824; RRID: Addgene_41824). AAVS1-TALEN-R was a gift from Danwei Huangfu (Addgene plasmid# 59026; http://n2t.net/addgene:59026; RRID: Addgene_59026). AAVS1-TALEN-L was a gift from Danwei Huangfu (Addgene plasmid# 59025; http://n2t.net/addgene:59025; RRID: Addgene_59025) [23]. gRNA sequences were obtained from GenScript gRNA database. 2mM Doxycyclin was used for inducible knockdowns.

Luciferase Assay

Cells were transfected with a SMAD2/3 reporter construct (SBE4-luciferase), and Renilla luciferase at a ratio of 10:1, using Lipofectamine 2000 (Invitrogen). Luciferase activity was measured with the dual luciferase assay kit following (Promega) manufacturer instructions. Firefly luciferase activity was normalized to Renilla luciferase activity for cell numbers and transfection efficiency. Samples were analysed on a Glomax Luminometer and software.

CRISPRi mediated knockdown

Cells were transfected with Dox-inducible CRISPR interference (CRISPRi) knock in construct into the AAVS1 locus. pAAVS1-NDi-CRISPRi (Gen2) was a gift from Bruce Conklin (Addgene plasmid # 73498; http://n2t.net/addgene:73498 ; RRID:Addgene_73498). Stable cell lines were differentiated with guide RNA construct pgRNA-CKB with a gRNA cloned into the construct. pgRNA-CKB was a gift from Bruce Conklin (Addgene plasmid # 73501; http://n2t.net/addgene:73501; RRID: Addgene_73501).

Transwell assays

Cancer cell invasiveness was analysed by using a modified Boyden chamber-based assay, CultureCoat 96 Well High BME Cell Invasion Assay (Trevigen, Cat. No: 3483-096-K) or 24-well tanswell inserts with 8µm pores (Sarstedt, Cat no. 83.3932.800) with EHS Matrix Extract as basal

membrane (Merck) according to manufacturer's guidelines. 25,000 cells were used per well and incubated for 24h before analysis. Cancer cells were placed in the media collected from their corresponding cell lines.

Nucleic acid extraction from cell lines

RNA was extracted using Direct-zol (TM) RNA extraction kit according to manufacturer protocol (Cambridge Bioscience, R2052). The quality of the RNA samples was verified using an RNA screen tape on a Tape-Station (Agilent). The RIN values for all samples were >7.5.

RNA Isolation and cDNA synthesis

Total RNA was isolated by RNeasy RNA Extraction Kit (Qiagen) according to manufacturer's guidelines. RNA was then eluted in 30µl of water and the concentration was measured using Nanodrop. The master mix was prepared as follows: 8µl 5x First-Strand Buffer (Invitrogen), 0.5µl Random primers (0.5 µg/ml) (Promega Cat. C1181), 1µl dNTP mix (10 mM each) (Promega Cat.U1515), 2 ul 0.1 M DTT, 0.5 µl RNase Out, 0.25 µl Superscript III Reverse Transcriptase (Life Technologies). 500 ng of total RNA into a separate tube with 11.75 µl RNase-free water. RNA was heated to 65 ºC for 5 min and allowed to chill on ice for 2 min. 8.25 µl of the master mix were added to RNA. The reaction was incubated at 25 ºC for 10 min and then at 42ºC for 50 min. The reaction was then inactivated by heating at 70 for 15 min.

RT-qPCR

2 ng of synthesized cDNA was added to 5µl Power SYBR Mix (Life Technologies, 4368708 (Master Mix)) and 1.5 µl 2µM of forward and reverse primers. RT-qPCR was performed on ViiA 7 machine with the following intervals: denaturation (95 ºC) for 15 s and a total of 40 cycles, annealing/extension (60 ºC) for 60s, final extension (60 ºC) for 10 minutes.

Flow cytometry for cell cycle analysis

A13A and FG PDAC cells in which the FUCCI construct was incorporated were taken from adherent conditions and counted, then plated in spheroid conditions at a density of 5,000 cells / 1 mL medium for 10 days, before the cells were collected and analysed using Fortessa (BD Bioscience). Passaging was performed a day 5, after which cells were plated again in spheroid conditions, with the same initial density of 5,000 cells / 1 mL medium. Compounds were added in day 7, and the treatment lasted for 72 hours, with 4 different conditions: I) I-BRD9 (10 µM); II) Gemcitabine (5 µM); III) I-BRD9

(10 μM) + Gemcitabine (5 μM); IV) DMSO control. Experiment was performed in 3 replicates. The data was analysed in FlowJo.

Preparation and Sequencing of Illumina RNA libraries

RNA-Seq libraries were created using the NEBNext Ultra RNA library prep kit using TruSeq indexes, following the manufacturer's recommendations. In summary, 500 ng of total RNA was used to isolate mRNA poly(A) by two rounds of purification using oligo dT magnetic beads followed by fragmentation and cDNA synthesis by random primers and reverse transcriptase. Bar-coded adapters were ligated to the cDNA fragments and a PCR reaction was performed to produce the sequencing libraries. To verify the library concentration and the library fragments length was used Agilent 2200 Tape-Station System. Adapter-ligated cDNA fragment libraries were sequenced on a NextSeq 500 platform (Illumina) using a paired-end run (2 × 41 bp).

RNA-sequencing analysis

Sequencing reads from the RNA-seq experiment were aligned to the human genome (hg38) using HISAT2 with the default parameters. FeatureCounts was used to assign mapped reads to genes with annotation gtf file Ensembl94. Differential gene expression analysis was performed using DESeq2 with IHW method for p value adjustment, apeglm method for effect size shrinkage, FDR-adjusted p value < 0.05 and log2 fold change > 0. Functional analysis of differential expressed genes was performed using Metacore. All plots were generated using R package 3.6.

Western blot analysis

Protein was isolated by lysing cells with RIPA Buffer (Sigma-Aldrich) supplemented by cOmplete EDTA-free protease inhibitor (Roche) and PhosSTOP™ (Sigma-Aldrich) and extracting the supernatant after high-speed centrifugation at 4°C. Protein quantification was performed using the Pierce BCA Protein Assay kit following the manufacturer's protocol. Isolated proteins were prepared for SDS-PAGE separation by dilution with 4× NuPAGE Sample buffer (Invitrogen), addition of NuPAGE™ Sample Reducing Agent ((10X), Invitrogen), 95°C for 5 minutes, and cooling. Isolated proteins were then analysed by Western blotting. Protein separation via SDS-PAGE was performed on a NuPAGE 4%–12% or 12% Bis-Tris gel (Life Technologies) with NuPAGE™ MOPS SDS Running Buffer (Life Technologies). Proteins were transferred to a PVDF membrane, blocked with 5% milk in PBS and 0.05% tween 20, probed with protein-specific antibodies, incubated with horseradish peroxidase-conjugated secondary antibodies, and visualized via enhanced chemiluminescence using the SuperSignal West Pico Chemiluminescent Substrate (Thermo

Scientific). All antibodies (Supplementary Figure 4) were diluted in 5% milk in PBS and 0.05% tween 20. Quantification was performed using ImageJ gel analysis tool.

Supplementary Table 3. Antibodies used in this study.

**Antibody raised against**	**Catalog number**	**Company**
Histone H3 (acetyl K27)	ab4729	Abcam
Histone H3 (tri methyl K36)	ab9050	Abcam
BRD9	ab137245	Abcam
SMAD2/3	AF3797	Bio-techne
SS18	GTX129428-S	Insight Biotechnology Ltd
BRG1 [GT2712]	GTX633391	Stratech Scientific Ltd
ARID1A/BAF250	A301-041A-T	Cambridge Bioscience Ltd
SMARCD1/BAF60a	A301-594A-T	Cambridge Bioscience Ltd
SMARCC2/BAF170	A301-038A-T	Cambridge Bioscience Ltd
SMARCC1/BAF155	A301-021A-T	Cambridge Bioscience Ltd
PBRM1/BAF180	A700-019-T	Cambridge Bioscience Ltd
BAF53A	A301-391A-T	Cambridge Bioscience Ltd
PHF10/BAF45A	66341-1-IG	Proteintech Europe Ltd
GLTSCR1	sc-515086	Insight Biotechnology Ltd
BCL11A (H-1)	sc-514842	Insight Biotechnology Ltd
Oct-3/4 (C-10)	sc-5279	Santa Cruz
Actin, clone C4	MAB1501	Chemicon
SOX4	C15310129	Diagenode
CD44	15675-1-AP	Proteintech
Ki67	ab15580	Abcam
Alexa Fluor 647 goat α-mouse IgM	A21238	Invitrogen
Alexa Fluor 647 donkey α-mouse IgG	A31571	Invitrogen
Alexa Fluor 647 donkey α-goat	A21447	Invitrogen

Immunostaining

The immunostaining method has been described previously [3-5]. Cells were fixed for 20 minutes at 4°C in PBS 4% PFA (electron microscopy grade), rinsed three times with PBS, and blocked and permeabilized at the same time for 30 minutes at room temperature using PBS with 10% Donkey Serum (Biorad) and 0.1% Triton X-100 (Sigma). Incubation with primary antibodies diluted in PBS 1% Donkey Serum 0.1% Triton X-100 was performed overnight at 4°C. Samples were washed three times with PBS, and then incubated with AlexaFluor secondary antibodies for 1 hour at room temperature protected from light. Cells were finally washed three times with PBS, and Hoechst

(Sigma) was added to the first wash to stain nuclei. Images were acquired using a LSM 700 confocal microscope (Leica).

Chromatin Immunoprecipitation (ChIP)

All steps were performed on ice or at 4°C and ice-cold buffers and PBS were supplemented with 1mg/ml Leupeptin, 0.2mM PMSF, and 10mM NaButyrate were used unless otherwise stated. Approximately $5x10^6$ cells were used per sample and cross-linked with 1% formaldehyde for 15 minutes. Cross-linking was stopped by incubating samples with glycine at a final concentration of 0.125M for 5 minutes at room temperature, and the cells were washed with PBS followed by pelleting at 250g for 5 minutes. The pellet was re-suspended in 2ml ChIP Cell Lysis Buffer (CLB: 10 mM Tris pH8, 10 mM NaCl, 0.2% NP-40) and incubated for 10 minutes to lyse the plasma membranes. Nuclei were pelleted at 600g for 5 min, lysed in 1.25ml of ChIP Nuclear Lysis Buffer (NLB: 50 mM Tris pH8, 10mM EDTA, 1% SDS) for 10 minutes, and then 0.75ml of ChIP Dilution Buffer (DB: 20 mM Tris pH8, 2mM EDTA, 150mM NaCl, 0.01% SDS, 1% Triton X-100) was added to the samples. Chromatin was sonicated in 15ml Diagenode Bioruptor Pico water bath sonicator with an automated water cooling system, by performing 30 cycles of 30 seconds ON, 45 seconds OFF. This protocol resulted in the homogeneous generation of fragments of 100-400bp. Samples were clarified by centrifugation at 16000g for 10 minutes, and diluted with 3.5ml of DB. After pre-clearing with 10µg of non-immune IgG for 1h and 50µl of Protein G-Agarose for 2h, ChIP was performed overnight in rotation using specific antibodies (Supplementary Table 2) or non-immune IgG as a control. After incubation for 1 hour with 30µl of Protein G-Agarose, beads were washed twice with ChIP Washing Buffer 1 (WB1: 20mM Tris pH8, 2mM EDTA, 50mM NaCl, 0.1% SDS, 1% Triton X-100), once with ChIP Washing Buffer 2 (WB2: 10mM Tris pH8, 1mM EDTA, 0.25M LiCl, 1% NP-40, 1% Deoxycholic acid), and twice with Tris-EDTA (TE: 10mM Tris pH8, 1mM EDTA). Precipitated DNA was eluted with 150µl of ChIP Elution Buffer (EB: 100mM $NaHCO_3$) twice for 15 minutes at room temperature in rotation, and processed as follows in parallel with 300 µl of sonicated chromatin non-used for ChIP (Input). Cross-linking was reverted by adding NaCl to a final concentration of 300mM for protein-DNA de-crosslinking and incubated at 65°C for 5 hours and 1 µg RNase A (Sigma) to digest contaminating RNA. Finally, 60 µg of Proteinase K (Sigma) were added overnight at 45°C. DNA was extracted by sequential phenol-chloroform and chloroform extractions, and precipitated overnight at -80°C in 100mM NaAcetate, 66% ethanol and 50µg of glycogen (Ambion) as a carrier. After centrifugation at 16,000g for 1 hour at 4°C, DNA pellets were washed once with ice-cold 70% ethanol, and finally air dried. ChIP samples were resuspended in 30µl and 1:10 of the samples were used in qPCR for verifying the ChIP samples.

Chromatin Immunoprecipitation (ChIP) sequencing.

Tagmentation was performed on ChIPed DNA by Tn5 transposase from Nextera DNA Sample Prep Kit (Illumina, cat. #FC-121-1031). Tagmented DNA purified by DNA Clean & Concentrator-5 kit (Zymo research, Cat# 4013). Tagmented DNA was subjected to PCR amplification and double-size selection by AMPure XP beads (Beckman, cat. #A63881) (0.55x/0.9x). The library was multiplexed, quantified using a High-sensitivity d1000 TapeStation (Agilent) and then sequenced using a NextSeq 500 (Illumina) (paired-end, 2 × 41 bp). Sequencing depth was >20 million reads per sample.

ATAC-sequencing

Cells were washed once with PBS, collected in Cell Dissociation Buffer (Gibco 13150-016) or TrypLE and centrifuged at 300g for 3 min. The cell pellets were then resuspended in 2 ml of 4°C PBS and counted by haemocytometer for using 100,000 cells in the subsequent step. Cells were centrifuged at 300g for 3 min, the supernatant aspirated, the cell pellet resuspended in 150 ul of Isotonic Lysis Buffer (10 mM Tris-HCl pH 7.5, 3 mM CaCl, 2 mM $MgCl_2$, 0.32 M Sucrose and Protease Inhibitors, Roche), and incubated for 12 min on ice. Triton X-100 from a 10% stock was then added at a final concentration of 0.5%, the samples were vortexed briefly and incubated on ice for 6 min. The samples were centrifuged for 5 min at 400g at 4°C, and the cytoplasmic fraction removed from the nuclear pellet. The samples were resuspended gently in 625uL of PBS and transferred to a fresh 1.5 ml eppendorf tube. The nuclei were centrifuged at 1500g for 3 min at 4°C and the supernatant aspirated thoroughly from the nuclear pellet. This step was immediately followed by tagmentation (Nextera DNA Sample Preparation Kit for 24 Samples, FC-121-1030) by resuspending each sample in 100 µL Nextera mastermix (52.5 µl TD buffer, 42.5 µl of water and 5 µl of TDE1 per reaction). The nuclear pellet was resuspended thoroughly by pipetting and incubated at 37 °C for 1 hour shaking at 300rpm. The reaction was stopped with 300 µL of buffer PB from the Qiagen PCR purification kit, followed by Qiagen PCR clean up protocol using MinElute columns and eluting each sample in 18 µl buffer EB. For the control sample, the nuclear pellet was subjected to genomic DNA isolation with GenElute Mammalian Genomic DNA Miniprep Kit (Sigma, G1N70) according to manufacturer's protocol, and the purified genomic DNA was thereafter immediately used for tagmentation as for other ATAC-seq samples.

Next a PCR reaction (for all samples including control sample) was performed with the following constituents: 10 µl template from tagmentation, 2.5 µl I7 primer (Nextera® Index Kit with 24 Indices for 96 Samples, FC-121-1011), 2.5 µl I5 primer, 10 uL Nudease Free H_2O 25µl NEBNext High-Fidelity 2x PCR Master Mix (New England Labs Cat #M054 and 10 uL Nuclease Free H_2O. The PCR settings were as follows: at 72 °C for 5 miutes, initial denaturation at 98 °C for 30 seconds, then 12 cycles of 98 °C for 10 seconds, primer annealing at 63 °C for 30 seconds and elongation at

72 °C for 1 minute, and holding at 4 °C. After completing the PCR, the sample were stored at -20 °C. The PCR primers were removed with 1 x 0.9:1 SPRI beads (Beckman Coulter, Cat no. A63880) according to manufacturer's protocol and samples eluted in 20 µl. 2 µl of the samples were run on Agilent HS Bioanalyzer HS for confirming the size selection of the ATAC libraries. ATAC-sequencing was performed by Illumina HiSeq 2000 sequencing with 75 bp PE for obtaining more than 40 million mapped reads per library. The raw and processed ATAC-seq data are publicly available on GEO (Accession number: GSE222952).

ATAC-sequencing analysis

Sequencing reads from the ChIP-seq and ATAC seq experiment were aligned to the human genome (hg38) using bowtie with reporting mode," –best –strata –v2". Deeptools was used to generate covergae track(bigwig). Coverage track was visualized by using UCSC genome browser. Peak calling was performed by using macs2 peak caller with default parameters for ChIP seq, and with parameter "--nomodel --shift -100 --extsize 200" for ATAC seq. Peaks annotated with nearest gene information by using BEDTools. Peak distribution over different genomic features were summarized by using Bioconductor package ChiPpeakAnno. Motif enrichment analysis within peak regions was performed using HOMER. All plots were generated using R package 3.6.

For the TF footprint analysis, motifs in JASPAR database were scanned using ATAC bam file and peak coordinates for Ctrl and IBRD9 separately. We then compared the cleavage profiles from Ctrl and IBRD9 for each TF: 2027 IBRD9 ATAC peaks within IBRD9 specific loop anchors and 2372 Ctrl ATC peaks within Ctrl specific loop anchors.

Single-cell RNA-sequencing on patient samples

Processing of the patient tumour samples

The Collagenase IV (Stem Cell Technologies, cat no: 7909) aliquot was defrosted in a water bath before sample collection. The freshly surgically removed PDAC sample was kept in the primary growth medium (500ml of DMEM/F12; 1:100 ml of L-glutamine; Pen-strep; 500ul of thermostable FGF2; 20ng/ml of EGF; 5ug/ml of Insulin; 1:50 of B27) after the surgery, and processed further inside a sterile Microbiological Safety Cabinet.

The tissue sample in the primary growth medium was poured onto a sterile 10cm petri dish and the sample was broken up into as small pieces as possible by using a scalpel. By using sterilised forceps, the tissue was collected into the 15ml falcon tube containing 10ml of primary growth medium + 2.5

mg/ml Collagenase IV + 2 mg/ml Dispase II (Sigma-Aldrich, Cat. no. 4942078001) + 1 mg/ml Trypsin Inhibitor (Sigma-Aldrich, Cat. no. T6522) + 1 unit/ml DNase I (NEB, Cat. no. M0303S) and incubated at 37°C with shaking speed at 50rpm for about 40 min. The dissociated cells were repeatedly collected at intervals of 20 min to increase cell yield and viability. Cell suspensions were filtered using a 70um cell strainer and red blood cells (RBC) were removed by RBC lysis buffer (Invitrogen, Cat. no. 1966634) with 1 unit/ml DNase I. The digested tissue was decanted into a 15ml falcon with growth medium and spun at 300xg for 10mins to sediment single cells. Cells were resuspended in primary growth media, counted (live/dead) and divided into four treatment conditions: i) DMSO. ii) I-BRD9 (Bio-Techne (R&D Systems), Cat no. 5591/10) at 10µM final concentration. iii) DMSO + Gemcitabine at 1µM final concentration. iv) I-BRD9 + Gemcitabine. Cells were transferred to low-adherent flat-bottomed plates. Cells were incubated with four different conditions for 72 hours. Cells were collected for single-cell RNA-sequencing.

Preparation of Single Cell Samples

After collection, the cells were centrifuged at a speed not exceeding 400rcf. The supernatant was discarded. The cell pellet was resuspended in 1mL 1X PBS containing 0.04% BSA and the washing procedure was repeated twice. After washing, appropriate volume PBS was added to the cell precipitation to obtain single-cell dispersion suspension with a concentration close to the goal number. A wide-bore pipette tip was used for pipetting cell resuspension for lower cell damage. 40µm Cell Strainers were used for removing cell debris and cell clumps. Automatic cytometry was used to determine the cell concentration. The sample volume was calculated based on the optimal cell sampling concentration supplied by the 10X official website and the target capture number. If the calculated concentration was too high, the liquid volume was adjusted to the appropriate concentration and the counting was repeated. Once the desired cell suspension was obtained, it was immediately placed on ice for subsequent GEMs preparation and reverse transcription.

Library Construction

The library construction was performed as follows: (1) Interrupt, end repair and add A base. Finally, the SPRI select beads were used to purify the product. (2) Adaptor ligation and SPRI select purification. (3) Index PCR and SPRI select purification. (4) Qubit® 3.0 Fluorometer (Life Technologies) was used to determine the library concentration. (5) Agilent 2100 High Sensitivity DNA Assay Kit (Agilent Technologies) was used to determine the distribution of library product fragments. After the library construction, Agilent 2100/LabChip GX Touch was used to detect the fragment length distribution of the library. Also, qPCR was used to accurately quantify the effective concentration of the library. The effective concentration of the library aimed > 10 nmol/L.

Single-cell gene expression analysis

Sequencing data was aligned and quantitated using CellRanger (10x Genomics) and hg38 reference genome sequence. Raw gene expression matrices were then analysed using Seurat R package (v4.0.5). Quality control was performed individually for each sample. All cells with <500 detected genes were removed, as well as cells contained <600 unique molecular identifiers (UMIs) and >10% mitochondrial counts. Raw read counts were normalized using *NormalizeData* function, based on which 2000 variable genes were identified. 2000 genes across samples for integration were selected using SelectIntegrationFeatures function. To perform data integration using reciprocal PCA method in FindIntegrationAnchors function, data scaling and PCA were performed on each sample first. 50 dimensions were used in integration process.

In order to identify cell types, we performed principal component analysis (PCA) on integrated data with default parameters in Seurat to reduce dimensionality. The first 50 principal components (PCs) were used for Uniform Manifold Approximation and Projection (UMAP) dimensionality reduction. The *FindNeighbours* (30 nearest neighbours) and *FindCluster* function (resolution = 2.5) were used to cluster cells. Clusters in 2D UMAP were used to identify cell types based on marker genes. Unless specified, default parameters were used for each function.

ChIP-sequencing data analysis

The adapters were removed and the filtered reads were mapped to hg38 reference genome using bwa [6] mem. The duplicated reads were removed using Picard (https://broadinstitute.github.io/picard/) and the reads with quality lower than 20 were filtered. Bam files were converted to bigwig format file using deepTools [7] bamCoverage. The parameter 'scaleFactor' for each sample were determined by edgeR [8] function calcNormFactors using "TMM" normalization method. The ChIP-seq peaks were called using the default (narrow) setting in MACS2. The promoter regions were defined as +/- 2.5Kb around the TSSs regions.

In-situ ChIA-PET and data analysis

Cells were grown to 80% confluency, and treated by 1% formaldehyde for 20 min and ethylene glycol bis (EGS) (Thermo Fisher Scientific, cat. #21565) for 45 min. 10 millions of dual crosslinked A13A CSC cells were used to generate one in-situ ChIA-PET library. Briefly, after cell lysis and nuclear lysis, cells were subjected to in-situ digestion with Alu I restriction enzyme for overnight. On the next day, A-tailing was performed using Klenow Fragment (3′>5′ exo-) (NEB, cat. #M0212L) and dATP (100 mM; NEB, cat.#N0440S) for 1 hr at room temperature. In-situ ligation was performed using in-

house bridge linker (F: 5′- /5Phos/CGCGATATC/iBIOdT/TATCTGACT -3′, R: 5′- /5Phos/GTCAGATAAGATATCGCGT -3′. HPLC purified, from Integrated DNA Technologies) and T4 DNA ligase (Thermo Fisher Scientific, cat. #EL0013) for RT 1 hr and then 16°C for overnight. The nucleus was sonicated into ~1 kb size. Then chromatin was precleared using protein G beads for 1 hr and immunoprecipitation was performed using anti-H3K27ac antibody (Active Motif, cat. #39133) coated protein G beads (Life Technologies, cat. no. 10009D) for overnight.

After that, proximity-ligated chromatin complexes were washed sequentially by low salt, high salt, LiCl buffer, and TE buffer. Chromatin complexes were eluted from the beads by incubation with ChIP elution buffer (1% SDS+TE) at 65 °C for 1 hr. After that, proteinase K (Life Technologies, cat. #AM2548) was added to the elution reverse cross-linked chromatin complexes for overnight. After DNA purification using QIAquick PCR Purification Kit, tagmentation was performed on the proximity-ligated DNA by Tn5 transposase from Nextera DNA Sample Prep Kit (Illumina, cat. #FC-121-1031). Then tagmented DNA was immobilized on M280 streptavidin dynabeads (Invitrogen, cat. #11205D). PCR amplification was performed on beads using Nextera DNA Sample Prep Kit and the products were purified by AMPure XP beads (Beckman, cat. #A63881) and subjected to size selection (350-600 bp) on BluePippin instrument (Sage Science) using Blue Pippin Cassette Kit (Sage Science, cat.#BDF2010). DNA library was sequenced on Illumina Novaseq 6000 by paired-end 150 bp.

In-situ **ChIA-PET data processing**

We used ChIA-PIPE [9] to process ChIA-PET data. Pair-end reads containing bridge linker were kept and trimmed, the flanking sequences were mapped to the human hg38 reference genome and uniquely aligned pair-end tags (PETs) with MAPQ $\geq$ 30 were retained. Redundant reads were then removed and bam format files were used for downstream analysis. Each PET was classified as self-ligation PET or inter-ligation PET using a genomic span cutoff of 8Kb. Inter-ligation PETs were extended by 500 bp on both ends and clustered. The PET clusters with counts lower than 10 and spanning less than 5 Kb or larger than 1Mb were filtered. We referred the remaining PET clusters as chromatin interaction loops. Un-clustered individual inter-ligation PETs were referred as PET singletons. All PETs (including self-ligation and inter-ligation PETs) were used to call peaks using MACS2 [10].

Chromatin interaction loops from all samples were merged to form a union set. Read counts for each merged loop were counted across all samples to form a loop – sample count matrix. To identify control-specific and IBRD9-specific loops, the read matrix was imported into R and a negative binomial model was fitted using glmFit function in edgeR [8] R package. The loops with p-value <0.1 were determined as differential loops [11]. Loop anchors proximal to (less than 3Kb) known

transcription start sites (TSSs) were defined as promoters and the remaining loop anchors were defined as distal regulatory elements.

Functional enrichment of downregulated genes with IBRD9 treatment

Functional enrichment of the genes network was generated using the ToppFun application of the Toppgene Suite using a corrected (Benjamini and Hochberg) p-value cut-off of 0.05 (https://toppgene.cchmc.org/) [12]. Cytoscape application (https://cytoscape.org/) [13] was used to generate the functional enrichment network.

Visualization

Statistical plots were created using R. Screenshots of chromatin interactions was visualized using the WashU Genome Browser.

Animal studies

All research protocols conformed to the 2011 Guidelines for the Care and Use of Laboratory Animals published by the National Institutes of Health. All animal use protocols and experiments were performed according to the guidelines approved by the Institutional Animal Care and Use Committee at Guangdong Medical Laboratory Animal Center and Yat-sen University. All NSG mice (Model Animal Research Center of Nanjing University) and PDX models (PDXM-221Pa, Shanghai Medicilon Inc) were maintained in a specific pathogen-free facility with high-efficiency particulate air (HEPA)-filtered air and given autoclaved food and water.

Establishment of xenograft tumour models in nude mice

The A13A cells for subcutaneous injection were divided into the following groups, 1) Null group, cells were transduced with scrambled shRNA adenovirus (Ad-GFP-U6-shScram, Vector Biolabs) containing a scramble sequence under the control of U6 promoter, with the GFP co-expression under a cytomegalovirus (CMV); 2) BRD9 KD group, cells were transduced with BRD9-targeted shRNA adenovirus (Ad-GFP-U6-shBRD9, Vector Biolabs); 3) Gemcitabine group, cells were treated with Gemcitabine (7.5 μM, Selleckchem, LY-188011); 4) BRD9 KD + Gemcitabine group, cells transduced with Ad-GFP-U6-shBRD9 were treated with Gemcitabine additionally. These cells were maintained at a low-density culture (1250 cells/ml, 312.5 cells/cm2) and harvested for subsequent experiments 72 h post transduction/treatment.

Tumour studies were performed in female 8-10 weeks old NSG mice. For tumour formation, 0.5×10^6 cells in 200µl of Matrigel/DMEM media mixture (1:1 ratio) were subcutaneously injected into the right flank of NSG mice.

Tumour growth and mouse survival

For tumour growth, mice were monitored twice a week or every 3 days by observation and palpation. Tumour size was measured with a digital caliper and tumour volume was calculated using the formular LxW2x0.5. Mouse body weight was measured twice weekly. Death, weight loss of 15% or more of body weight, tumour volume with 2,000m^3 or more, or severe labored breathing or dyspnea were considered endpoints and all mice were euthanized.

In vivo PET imaging

Mice were fasted for 12 h before 18F- fluorodeoxyglucose (FDG) injection but allowed free access to water. After anesthesia with 2% isoflurane, mice were injected with 18F-FDG (11 to 16MBq, German Cancer Research Center, Heidelberg, Germany) by tail vein and kept at 37oC until imaging. Imaging was started at 60 minutes after 18F-FDG injection with non-invasive micro-positron emission tomography (µPET, Siemens Inveon). A CT scan (80 kVp, 500 µA, at 120 projections; approximately 4 minutes) was acquired for anatomical reference and enabled PET attenuation correction during reconstruction. Postprocessing was carried out with Inveon Research Workplace.

Therapeutic studies in PDX models

Therapy was initiated in PDX mice after the tumour reached an average volume of 150mm^3 with a repeated injection schedule (once every 3 days for a total of six treatments within 15 days). The PDX mice with various treatments were divided into the following groups, 1) Control treatment group (Ctrl), the mice were administrated with DMSO in the same manner as the treatment groups; 2) Gemcitabine treatment group (Gem), the mice were administrated with Gemcitabine (50 mg/kg i.p.); 3) Combination of BRD9 inhibitor with Gemcitabine (IBRD9 + Gem), the mice were administrated with Gemcitabine (25 mg/kg i.p.) and BRD9 inhibitor (10 mg/kg i.p.).

Histology analysis with H&E staining

Mouse tumour tissues were dissected, rinsed with PBS, and fixed in 10% formalin for 48 h. Following dehydration through a series of ethanol solutions, the tissues were embedded in paraffin wax according to standard laboratory procedures. Subsequently, they were sectioned (5 µm thick) using a microtome. Following deparaffinization and rehydration, H&E staining was performed on the sections. In brief, the sections were stained in Mayers Hematoxylin for 1 min. Following rinsing in

tape water, the sections were stained in Alcoholic-Eosin for 1 minute and dehydrated and cleared with xylene. The images were examined with an Olympus BX41 microscope equipped with a CCD (Magna-Fire TM) camera.

Immunohistochemistry assay

After antigen retrieval in sodium citrate buffer (10mM Sodium Citrate, 0.05% Tween 20, pH 6.0), the paraffin-embedded or frozen tumour sections were incubated with different primary antibodies: Ki67, (1:200), CD44 (1:200), and SOX2 (1:200) in 0.02% Triton for overnight at 4°C. After triple washing in PBS, slides were incubated for 45 min at 37 °C with different fluorescence-conjugated second antibodies (Jackson Immuno Research). DAPI was used for nuclear counterstaining. Four fields of each section were examined for quantification. Fluorescent imaging was performed with an Olympus BX41 microscope equipped with an epifluorescence attachment.

ChIP-qPCR of anchor regions

The cells were treated as for RNA-sequencing experiment and the ChIP was performed as described above. The primers used for ChIP-qPCR of anchor regions are listed in Supplementary Table 5.

Supplementary Table 4. ChIP-qPCR primers of anchor regions.

**Primer name**	**Primer sequence**
SOX4 enhancer anchor F	TATGTTTGGCCAGCTGTTGA
SOX4 enhancer anchor R	GGAGAAAGAGGTAAGAATGATCAGA
PROM1 enhancer anchor F	TCTTATTGCAGGGTCTGAGC
PROM1 enhancer anchor R	TTGTTCAGTAAATGAATACATGAAAGA
SMAD1 enhancer anchor F	CTTCCACATCAAGGATGAGC
SMAD1 enhancer anchor R	GCAATATAACTGGGCTGCAC
SNAI2 enhancer anchor F	CACTTGACTGGCTTCCAAGG
SNAI2 enhancer anchor R	AAGGAGGGTGGAGCATGATT

3C-qPCR

The 3C-qPCR assay was conducted with modifications to a previously published method (Hagège et al 2007). Briefly, 5 million cells were fixed with 2% formaldehyde for 10 minutes at room temperature and subsequently lysed. In-situ digestion was performed using restrict enzyme AluI (New England Biolabs), followed by ligation with T4 ligase (T4 ligase, New England Biolabs). The design of the 3C-qPCR primers employed a unidirectional strategy. Specifically, the primers were

designed on the "forward" strand, positioned near the AluI restriction site (AGCT), within a range of 20 to 100 base pairs. Loading control primers for GAPDH were designed on the AluI fragment that does not overlap with the AluI restriction site. The primers for 3C-qPCR are as Supplementary Table 5.

Supplementary Table 5. 3C-qPCR primers.

hg38		
	**Coordinate**	**Primer sequence**
SOX4	chr6:21594089-21594108	CGCTCGTGAACTGGAATCAA
	chr6:21949888-21949907	CCGTGAATGTATTCGTCCAC
PROM1	chr4:16084018-16084037	GGATCTGCCTCAGTCACTTA
	chr4:16129495-16129514	TATTTGTTGCCCTGCTGTGG
SMAD1	chr4:145482992-145483011	ATGTGCTTTAGGAGGGGAGG
	chr4:145279604-145279623	TGTGACAGAAGCCTTGGTCC
SNAI2	chr4:48788764-48788784	GTGGTCAGAGTGAGAGGTTCA
	chr4:48921569-48921588	TCTGGTTCAAAATGGGCTGT
GAPDH	chr12:6534816-6534835	CACATCGCTCAGACACCATG
	chr12:6534935-6534954	CATACGACTGCAAAGACCCG

Supplementary References

e1 Krentz, N. A., Nian, C. & Lynn, F. C. TALEN/CRISPR-mediated eGFP knock-in add-on at the OCT4 locus does not impact differentiation of human embryonic stem cells towards endoderm. *PloS one* **9**, e114275, doi:10.1371/journal.pone.0114275 (2014).

e2 Hockemeyer, D. *et al.* Genetic engineering of human pluripotent cells using TALE nucleases. *Nat Biotechnol* **29**, 731-734, doi:10.1038/nbt.1927 (2011).

e3 Pauklin, S., Madrigal, P., Bertero, A. & Vallier, L. Initiation of stem cell differentiation involves cell cycle-dependent regulation of developmental genes by Cyclin D. *Genes Dev* **30**, 421-433, doi:10.1101/gad.271452.115 (2016).

e4 Pauklin, S. & Vallier, L. The cell-cycle state of stem cells determines cell fate propensity. *Cell* **155**, 135-147, doi:10.1016/j.cell.2013.08.031 (2013).

e5 Bertero, A. *et al.* Activin/nodal signaling and NANOG orchestrate human embryonic stem cell fate decisions by controlling the H3K4me3 chromatin mark. *Genes Dev* **29**, 702-717, doi:10.1101/gad.255984.114 (2015).

e6 Li, H. & Durbin, R. Fast and accurate short read alignment with Burrows-Wheeler transform. *Bioinformatics* **25**, 1754-1760, doi:10.1093/bioinformatics/btp324 (2009).

e7 Ramirez, F., Dundar, F., Diehl, S., Gruning, B. A. & Manke, T. deepTools: a flexible platform for exploring deep-sequencing data. *Nucleic Acids Res* **42**, W187-191, doi:10.1093/nar/gku365 (2014).

e8 Robinson, M. D., McCarthy, D. J. & Smyth, G. K. edgeR: a Bioconductor package for differential expression analysis of digital gene expression data. *Bioinformatics* **26**, 139-140, doi:10.1093/bioinformatics/btp616 (2010).

e9 Lee, B. *et al.* ChIA-PIPE: A fully automated pipeline for comprehensive ChIA-PET data analysis and visualization. *Sci Adv* **6**, eaay2078, doi:10.1126/sciadv.aay2078 (2020).

e10 Zhang, Y. *et al.* Model-based analysis of ChIP-Seq (MACS). *Genome Biol* **9**, R137, doi:10.1186/gb-2008-9-9-r137 (2008).

e11 Mumbach, M. R. *et al.* Enhancer connectome in primary human cells identifies target genes of disease-associated DNA elements. *Nature genetics* **49**, 1602-1612, doi:10.1038/ng.3963 (2017).

e12 Chen, J., Bardes, E. E., Aronow, B. J. & Jegga, A. G. ToppGene Suite for gene list enrichment analysis and candidate gene prioritization. *Nucleic Acids Res* **37**, W305-311, doi:10.1093/nar/gkp427 (2009).

e13 Shannon, P. *et al.* Cytoscape: a software environment for integrated models of biomolecular interaction networks. *Genome Res* **13**, 2498-2504, doi:10.1101/gr.1239303 (2003).

Supplementary Figures

Supplementary Figure 1. BRD9 inhibition by small molecules or knockdown reduces CSCs. (**A**) Establishing the cell line platform for small molecule screening. eGFP ORF was inserted in frame at the 3’ region of the endogenous *OCT4* locus via TALEN-mediated recombination and selected for the insertion with Puromycin that is inserted with eGFP under the control of the PGK promoter. The insertion of eGFP into the OCT4 locus of FG cells was validated by PCR amplification of 5’ and 3’ regions by using primers covering the OCT4 locus and the eGFP sequence in the correct orientation. (**B**) TGFβ/Activin signaling induces chemoresistant OCT4-GFP+/CD133+/SSEA4+ CSCs. Cells expressing CSC markers OCT4-GFP, CD133 and SSEA4 are more resistant to Gemcitabine, 5-FU and Paclitaxel treatment than CSC marker negative cancer cells. FG cells were treated with 0.5μM

Gemcitabine, 3µM 5-FU, and 0.5µM Paclitaxel in combination with Activin A or 10 µM SB431542 for 5 days, followed by flow cytometry analysis of CSC markers OCT4-GFP, CD133 and SSEA4. Cells were gated for DAPI negative signal for live cells. While most cancer cells are killed by the chemotherapy reagents, the DAPI negative surviving cells are enriched for cancer stem cell markers. (**C**) TGFβ/Activin-SMAD2/3 signaling improves cell survival upon the treatment with chemotherapy reagents. Cells were treated with Gemcitabine, 5-FU and Paclitaxel as in (**B**). (**D-E**) Higher expression of CD133/PROM1 (p-value=0.0292) and OCT4 (p-value=0.135) correlates with lower survival of pancreatic cancer patients according to TCGA data (**D**), and lower disease-free survival (**E**). (**F**) Self-renewal of CSC marker positive and negative cell populations. (**G**) 2-fold serial dilution assay of CSC marker positive and negative cell populations. (**H-J**) The expression of CSC markers CD133, OCT4, and SSEA4 on live PDAC cells. (H) Representative dot plots in DMSO control (upper panels) and Gemcitabine treatment (lower panels). (I) Percentage of CSC marker double-positive cells. (J) Percentage of CSC marker triple-positive cells. (**K**) dBRD9 PROTAC mediated BRD9 protein degradation. Western blotting of BRD9 indicates the reduction of BRD9 protein level upon 10µM or 50µM dBRD9 treatment for 24h or 72h. (**L**) Schematic depiction of BRD9 PROTAC that leads to the degradation of BRD9 protein. (**M-P**) BRD9 stable knockdown clone validation at the mRNA level in FG and A13A cells (M, O) and protein level by western blotting in FG and A13A cells (N, P). (**R**) Relative change in OCT4+/CD133+/SSEA4+ triple positive cells upon Bromodomain inhibitor treatments. (**S**) List of Bromodomain inhibitors with their targets that showed an effect on the relative abundance of OCT4+/CD133+/SSEA4+ triple positive cells during the compound screening process. (**T**) BRD1, BRD4 and SMARCA2/4 inhibition decreases CSC self-renewal. Statistical analysis was performed by 2-way ANOVA with multiple comparisons with Tukey correction and **** marks adjusted P-value <0.0001, *** is adjusted P-value <0.001, ** is adjusted P-value <0.01, * is adjusted P-value <0.05.

Supplementary Figure 2. BRD9 inhibition reduces several non-overlapping heterogenous CSC populations. (**A-B**) BRD9 inhibition in PDAC cell lines reduces the relative abundance of CD44, ALDH, CXCR4, DCLK1, and MDR1 positive cells shown in (A) representative dot plots of FG cell treatment and in (B) bar graphs. (**C-D**) BRD9 inhibition in primary patient-derived PDAC samples reduces the relative abundance of CD44, ALDH, CXCR4, DCLK1, and MDR1 positive cells. Statistical analysis was performed by 2-way ANOVA with multiple comparisons with Tukey correction and **** marks adjusted P-value <0.0001, *** is adjusted P-value <0.001, ** is adjusted P-value <0.01, * is adjusted P-value <0.05.

Supplementary Figure 3. BRD9 inhibition sensitizes PDAC cells to chemotherapy treatment. (**A-B**) BRD9 depletion/inhibition in standard 2D culture conditions slows cell proliferation in FG (A) and A13A cells (B). (**C**) BRD9 knockdown sensitizes PDAC cells to Gemcitabine treatment in 2D growth conditions. Cells were treated with 0.1µM Gemcitabine for 5 days. (**D**) SMARCA2/4 protein degradation with protac AU-15330 in PDAC cell lines. (**E**) SMARCA2/4 protein degradation or inhibition reduces CSC self-renewal and increases chemosensitivity in PDAC cell lines. (**F**) TGFβ/Activin signaling inhibition by 10 µM SB431542 for 5 days eliminates OCT4-GFP+/CD133+/SSEA4+ CSCs. FG cells were treated with 10 µM SB431542 for 5 days, followed by flow cytometry analysis of CSC markers OCT4-GFP, CD133 and SSEA4. (**G**) The percentage of CSC marker positive cells is reduced by I-BRD9 or SB431542. (**H**) TGFβ/Activin signaling inhibition and BRD9 inhibition impair CSC self-renewal and sensitize CSCs for Gemcitabine-mediated destruction in FG cells. (**I**) BRD9 inhibition sensitizes PDAC cells for elimination by Gemcitabine. A13A cells were treated for 5 days and quantified for cell numbers. (**J-K**) BRD9 knockdown abolishes sphere formation by sensitizing them for Gemcitabine and 5-FU treatment in FG cells (**J**) and A13A (**K**). (**L**) BRD7 knockdown in A13A, FG and L3.6pl cell lines. (**M**) BRD9 incorporation into the BAF complex increased in BRD7 knockdown cells. Co-IP experiments show increased association of BRD9 with BAF155 in BRD7 knockdown cells. (**N**) BRD7 knockdown modestly increases the self-renewal capacity of CSCs. (**O**) BRD9 binding to SOX4, PROM1, SNAI2, and SMAD1 loci is modestly increased upon BRD7 knockdown. Statistical analysis was performed by 2-way ANOVA with multiple comparisons with Tukey correction and **** marks adjusted P-value <0.0001, *** is adjusted P-value <0.001, ** is adjusted P-value <0.01, * is adjusted P-value <0.05.

Supplementary Figure 4. BRD9 inhibition impacts G0 phase entry of CSCs and forms a protein complex with SMAD2/3. (**A-B**) Cell cycle analysis with the FUCCI system in (A) A13A and (B) FG cells, treated with I-BRD9, Gemcitabine or the dual treatment with Gemcitabine and I-BRD9 for 5 days. (**C-D**) Flow cytometry analysis of the FUCCI signal and density blots of (C) A13A and (D) FG cells. (**E**) BRD9 loss-of-function reduces cell migration in trans-well assays. (**F**) The protein interaction network based on STRING database analysis depicting SMAD2/3 interaction with the subunits of the BAF protein complex. (**G**) Schematic depiction of the different subunit composition of non-canonical BAF, esBAF, and npBAF. SMAD2/3 proteins interact with the subunits of non-canonical BAF, esBAF, and npBAF complexes in pancreatic CSCs. Co-immunoprecipitation of unique subunits that are specific for esBAF (BCL11a), npBAF (BAF180) and ncBAF (GLTSCR1).

Supplementary Figure 5. Inhibition of BRD9 abolishes PDAC tumour growth in mice. (**A**) Schematic depiction of the mouse experiments. (**B**) Tumour volume measurements indicate reduced tumour sized upon BRD9 knockdown. (**C**) PET imaging of tumours in mice indicates reduced tumour

sizes upon BRD9 knockdown and absence of visible tumours upon BRD9 knockdown with Gemcitabine treatment. The images show PET scan in 2D and in 3D with the tumour highlighted in yellow dotted areas. (**D**) Tumour size comparison in different treatments. Tumours from non-treated cells are larger compared to Gemcitabine-treated and BRD9 knockdown PDAC cells, whereas BRD9 knockdown with Gemcitabine treatment resulted in undetectable tumours at 3 months of growth. (**E**) Relative tumour weight measurements in the different treatment conditions. (**F**) Histological analysis of tumours. N=necrotic areas.

Supplementary Figure 6. BRD9 inhibition eliminates CSCs in patient-derived primary PDAC tumour cells. (**A**) Enriched GO terms for genes that are downregulated in I-BRD9 treated and I-BRD9/Gemcitabine treated samples compared to DMSO and Gemcitabine treated samples. (**B**) Enriched Pathway terms for genes that are downregulated in I-BRD9 treated and I-BRD9/Gemcitabine treated samples compared to DMSO and Gemcitabine treated samples. (**C**) BRD9 inhibition leads to the elimination of cells that are enriched in CSC population such as JUN, SOX4, and SKIL. Expression levels of JUN, SOX4, and SKIL in the CSC population are shown as dot plot graphs where blue color indicates higher expression level compared to gray colour. (**D**) Heatmap of the lost and gained CREs upon BRD9 inhibition. (**E**) BRD9 inhibition reduces the expression of *SOX4*, *TWIST1*, *SNAIL2*, *JUN*, *IL6* and *IGF1* in CSCs at the mRNA level. Statistical analysis was performed by 2-way ANOVA with multiple comparisons with Tukey correction and **** marks adjusted P-value <0.0001, *** is adjusted P-value <0.001, ** is adjusted P-value <0.01, * is adjusted P-value <0.05.

Supplementary Figure 7. IBRD9 treatment reduces stemness factors and EMT regulators. (**A-B**) BRD9 inhibition leads to the elimination of cells that expressed in CSC population such as self-renewal factors *HOXA4*, *HOXA5*, *HOX3A*, *YAP1*, *MSI2*, and *NOTCH2* (A), and EMT regulators *SNAIL1*, *SNAIL2*, *TWIST2*, *ZEB1* and *ZEB2* (B). Expression levels of genes in the CSC population are shown as dot plot graphs where the blue color indicates higher expression level compared to the gray color. (**C**) Populations of cells changing under IBRD9 treatment overlap with gene expression signatures of the basal subtype of PDACs and an EMT signature. Sig.2 and sig.10 mark basal-like subset which is linked to bad prognosis and bad chemotherapy response; sig.1 and sig.6 mark classical subset, which suggests a better prognosis.

Supplementary Figure 8. BRD9 inhibition changes the enhancer-promoter connectome of CSCs. (**A**) GSEA analysis of genes regulated by BRD9 inhibition indicate the enrichment of genes involved in epithelial-to-mesenchymal transition, cell migration, regulation of cell population

proliferation, and vasculature development. (**B-C**) BRD9 inhibition leads to the loss of enhancer-promoter looping and loss of expression at *SMAD1* locus (B), and *LDHA* and *PNPA7*. The genomic loci show H3K27ac abundance and gene transcription together with 3D chromatin interactions. (**D**) P-SMAD3 levels do not change upon I-BRD9 treatment in A13A PDAC cells. (**E**) Nuclear levels of SMAD2/3 protein do not change upon I-BRD9 treatment in A13A PDAC cells. (**F**) Global SMAD2/3-dependent transcriptional activity is not affected by I-BRD9 treatment. (**G**) IBRD9 reduces the expression of *SNAI2*, *SNAI1*, and *TWIST2* in a locus-specific way. (**H**) DPY30 and CBP binding to BRD9 loci depend on TGFβ/Activin signaling. Statistical analysis was performed by 2-way ANOVA with multiple comparisons with Tukey correction and **** marks adjusted P-value <0.0001, *** is adjusted P-value <0.001, ** is adjusted P-value <0.01, * is adjusted P-value <0.05.

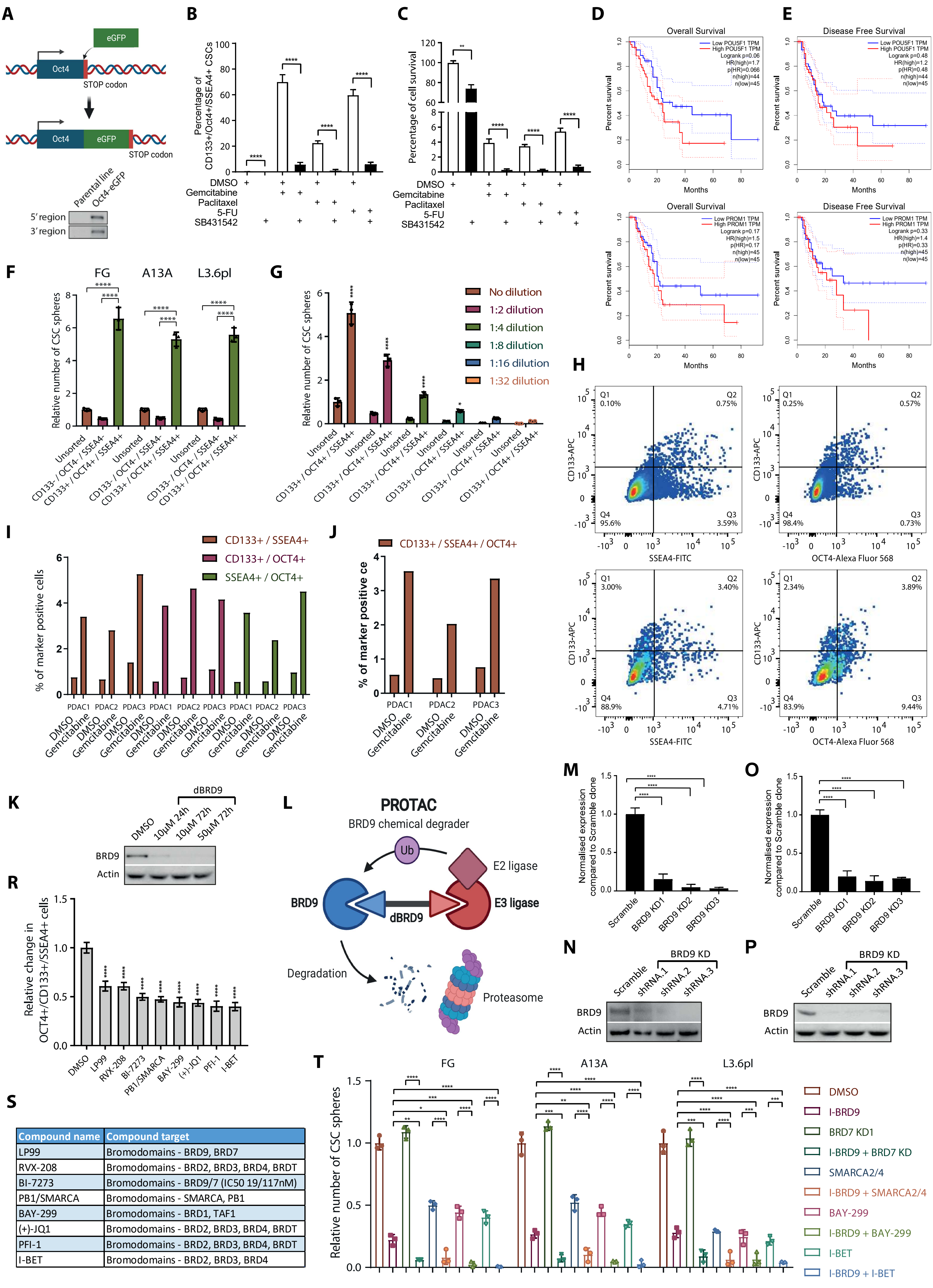

Compound name	Compound target
LP99	Bromodomains - BRD9, BRD7
RVX-208	Bromodomains - BRD2, BRD3, BRD4, BRDT
BI-7273	Bromodomains - BRD9/7 (IC50 19/117nM)
PB1/SMARCA	Bromodomains - SMARCA, PB1
BAY-299	Bromodomains - BRD1, TAF1
(+)-JQ1	Bromodomains - BRD2, BRD3, BRD4, BRDT
PFI-1	Bromodomains - BRD2, BRD3, BRD4, BRDT
I-BET	Bromodomains - BRD2, BRD3, BRD4

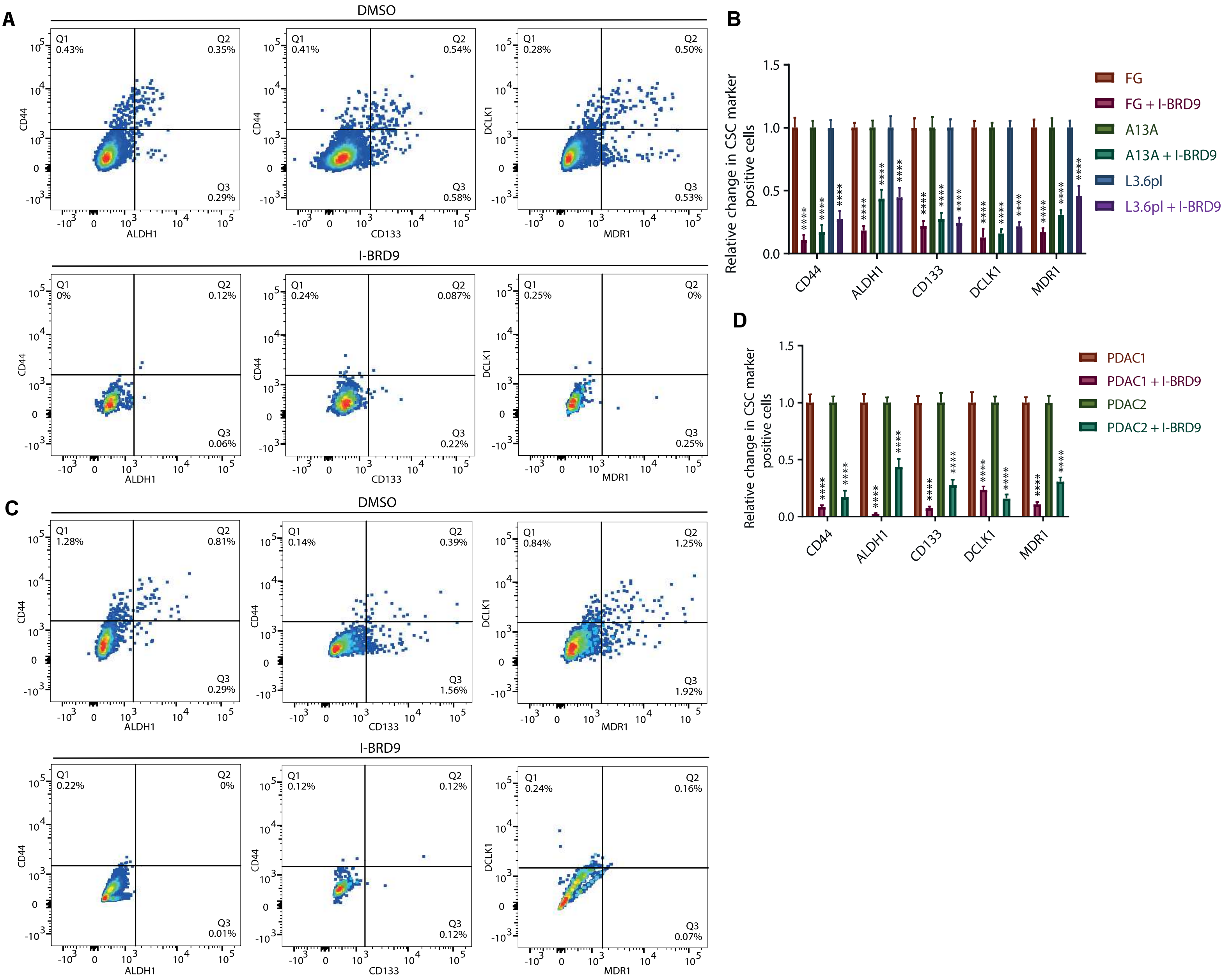

A
DMSO
Q1 0.43%
Q2 0.35%
Q3 0.29%
Q1 0.41%
Q2 0.54%
Q3 0.58%
Q1 0.28%
Q2 0.50%
Q3 0.53%
CD44
DCLK1
ALDH1
CD133
MDR1
I-BRD9
Q1 0%
Q2 0.12%
Q3 0.06%
Q1 0.24%
Q2 0.087%
Q3 0.22%
Q1 0.25%
Q2 0%
Q3 0.25%
B
Relative change in CSC marker positive cells
FG
FG + I-BRD9
A13A
A13A + I-BRD9
L3.6pl
L3.6pl + I-BRD9
CD44
ALDH1
CD133
DCLK1
MDR1
****
C
DMSO
Q1 1.28%
Q2 0.81%
Q3 0.29%
Q1 0.14%
Q2 0.39%
Q3 1.56%
Q1 0.84%
Q2 1.25%
Q3 1.92%
I-BRD9
Q1 0.22%
Q2 0%
Q3 0.01%
Q1 0.12%
Q2 0.12%
Q3 0.12%
Q1 0.24%
Q2 0.16%
Q3 0.07%
D
Relative change in CSC marker positive cells
PDAC1
PDAC1 + I-BRD9
PDAC2
PDAC2 + I-BRD9

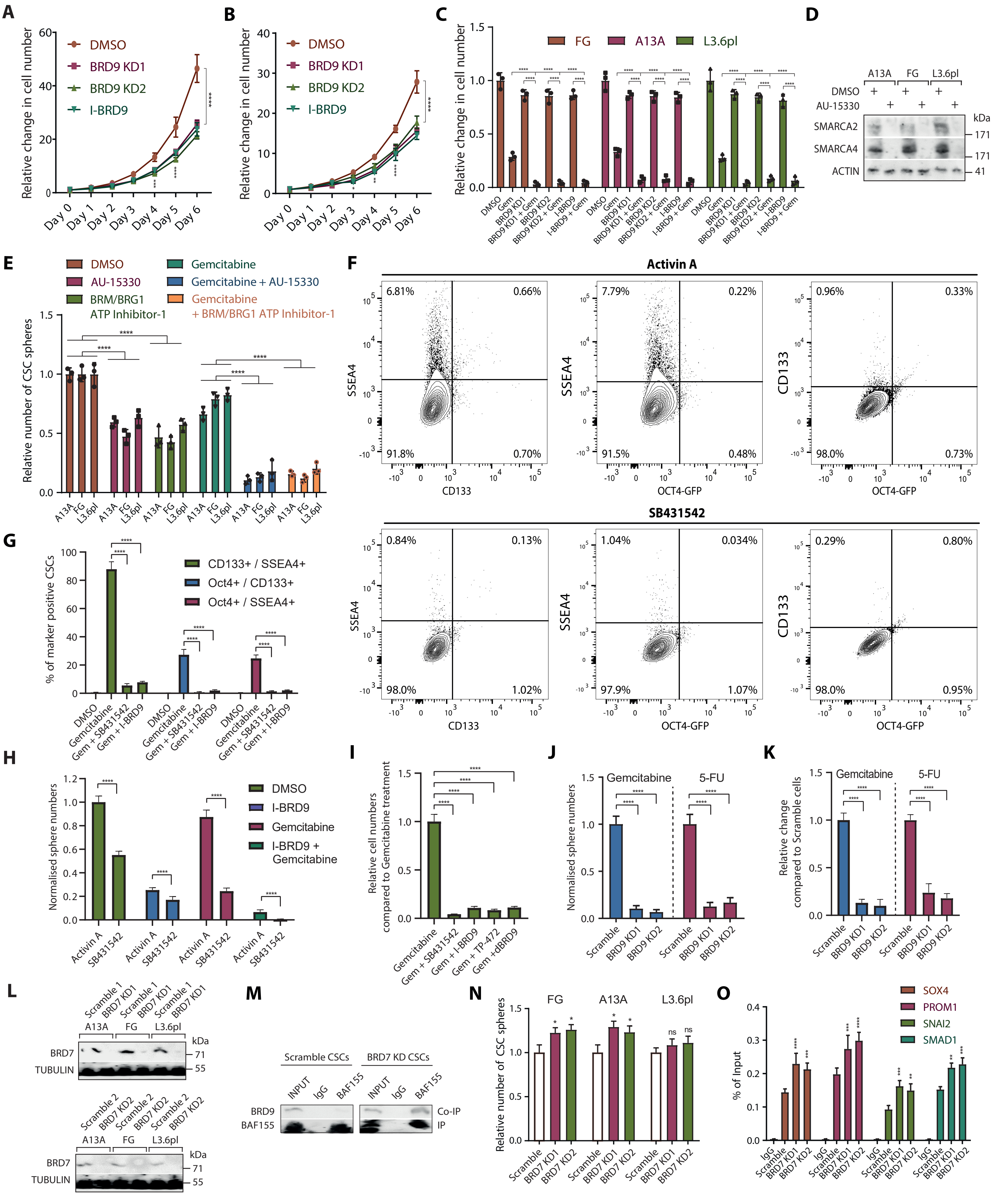
A
B
C
D
E
F
G
H
I
J
K
L
M
N
O
DMSO
BRD9 KD1
BRD9 KD2
I-BRD9
Relative change in cell number
FG
A13A
L3.6pl
DMSO
AU-15330
SMARCA2
SMARCA4
ACTIN
kDa
AU-15330
Gemcitabine
Gemcitabine + AU-15330
BRM/BRG1 ATP Inhibitor-1
Gemcitabine + BRM/BRG1 ATP Inhibitor-1
Relative number of CSC spheres
Activin A
SB431542
SSEA4
CD133
OCT4-GFP
CD133+ / SSEA4+
Oct4+ / CD133+
Oct4+ / SSEA4+
% of marker positive CSCs
Normalised sphere numbers
Gemcitabine
I-BRD9 + Gemcitabine
Relative cell numbers compared to Gemcitabine treatment
5-FU
Scramble
Relative change compared to Scramble cells
BRD7
TUBULIN
Scramble CSCs
BRD7 KD CSCs
INPUT
IgG
BAF155
BRD9
Co-IP
IP
BRD7 KD1
BRD7 KD2
SOX4
PROM1
SNAI2
SMAD1
% of Input

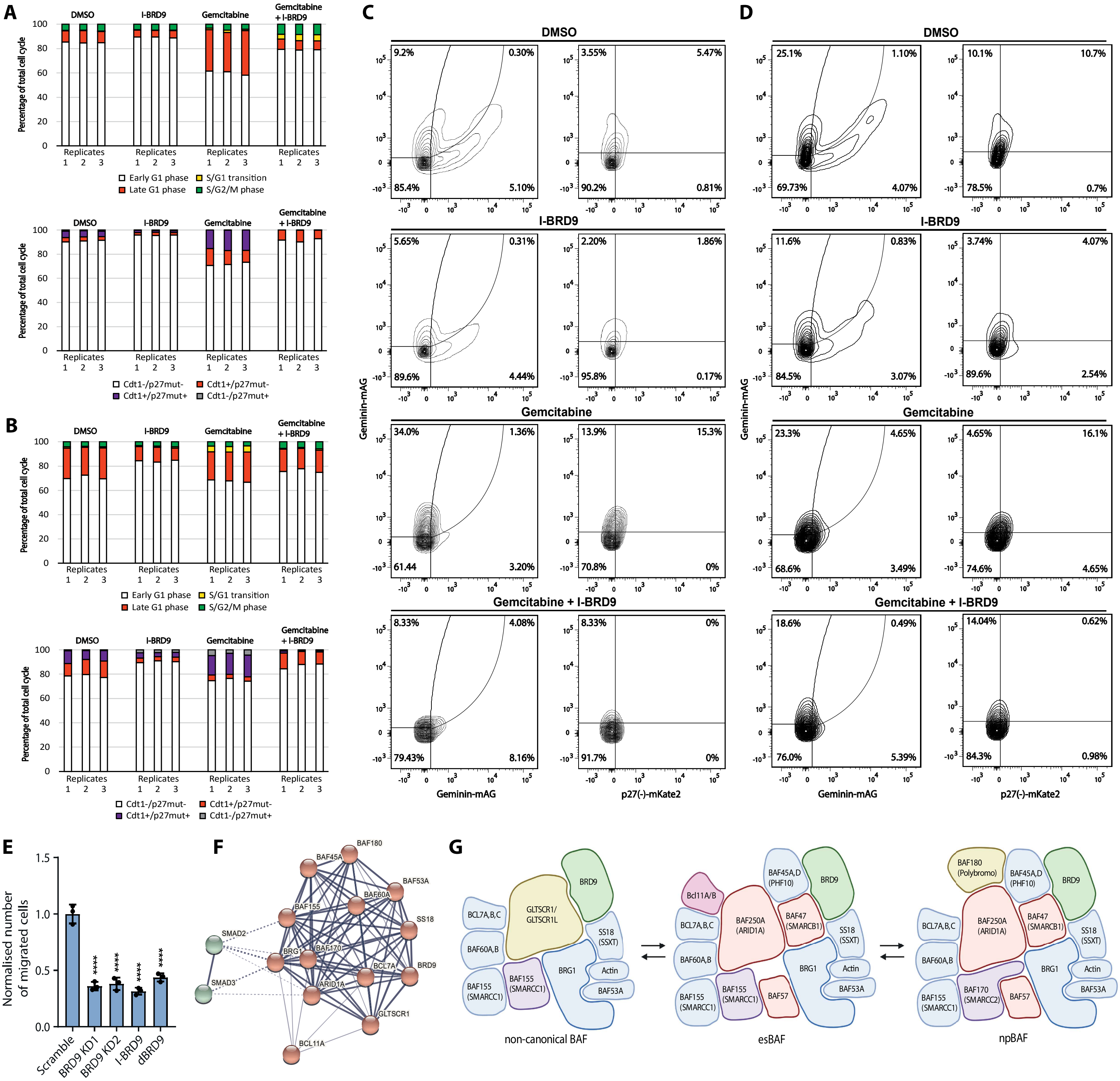

A
B
C
D
E
F
G
DMSO
I-BRD9
Gemcitabine
Gemcitabine + I-BRD9
Percentage of total cell cycle
Replicates
Early G1 phase
Late G1 phase
S/G1 transition
S/G2/M phase
Cdt1-/p27mut-
Cdt1+/p27mut-
Cdt1+/p27mut+
Cdt1-/p27mut+
Geminin-mAG
p27(-)-mKate2
Normalised number of migrated cells
Scramble
BRD9 KD1
BRD9 KD2
I-BRD9
dBRD9
non-canonical BAF
esBAF
npBAF

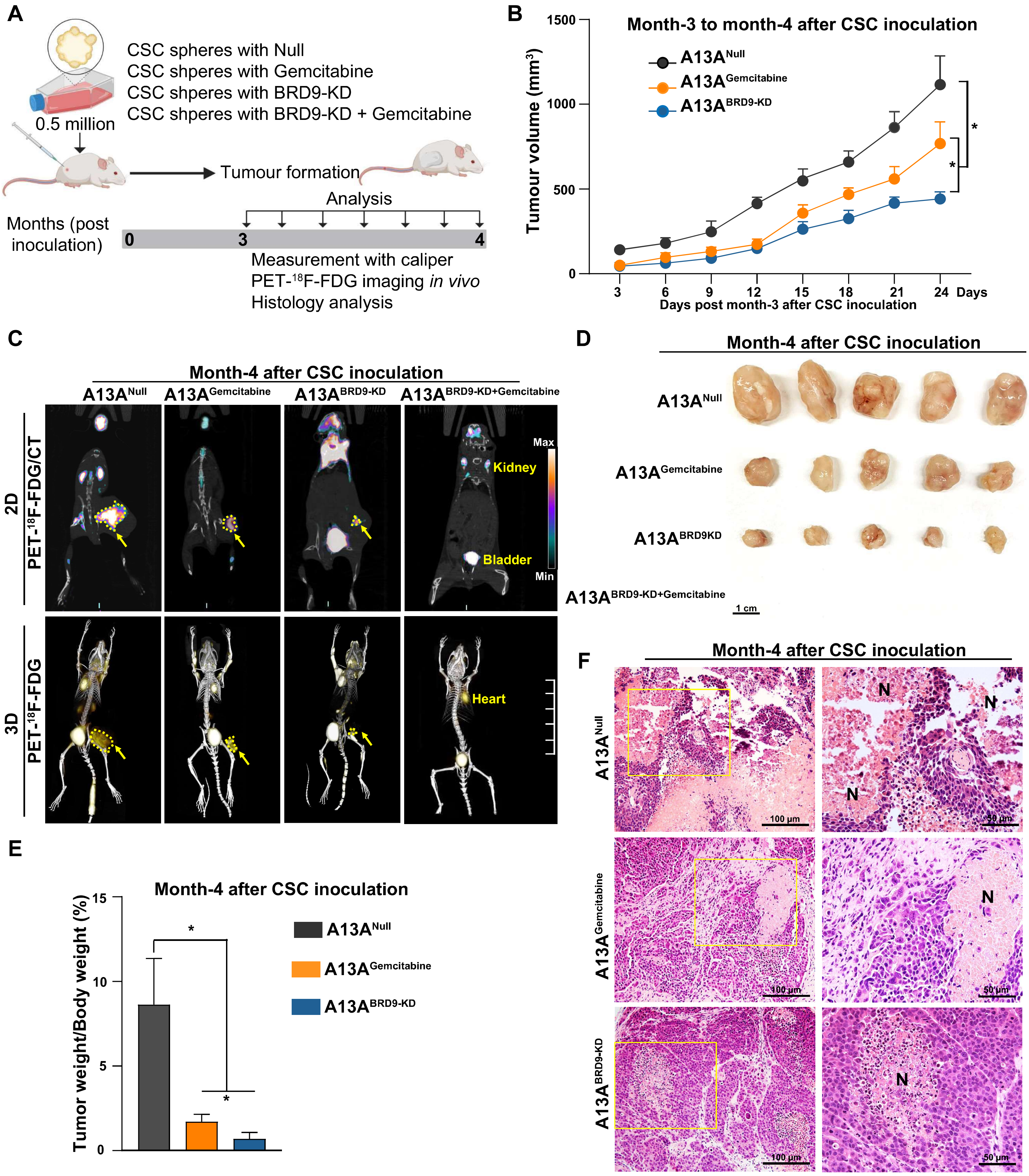
A
CSC spheres with Null
CSC shperes with Gemcitabine
CSC shperes with BRD9-KD
CSC shperes with BRD9-KD + Gemcitabine
0.5 million
Tumour formation
Analysis
Months (post inoculation)
0
3
4
Measurement with caliper
PET-18F-FDG imaging in vivo
Histology analysis
B
Month-3 to month-4 after CSC inoculation
A13A Null
A13A Gemcitabine
A13A BRD9-KD
Tumour volume (mm3)
Days post month-3 after CSC inoculation
Days
C
Month-4 after CSC inoculation
A13A Null
A13A Gemcitabine
A13A BRD9-KD
A13A BRD9-KD+Gemcitabine
2D PET-18F-FDG/CT
3D PET-18F-FDG
Max
Min
Kidney
Bladder
Heart
D
Month-4 after CSC inoculation
A13A Null
A13A Gemcitabine
A13A BRD9KD
A13A BRD9-KD+Gemcitabine
1 cm
E
Month-4 after CSC inoculation
Tumor weight/Body weight (%)
A13A Null
A13A Gemcitabine
A13A BRD9-KD
F
Month-4 after CSC inoculation
A13A Null
A13A Gemcitabine
A13A BRD9-KD
N
100 µm
50 µm

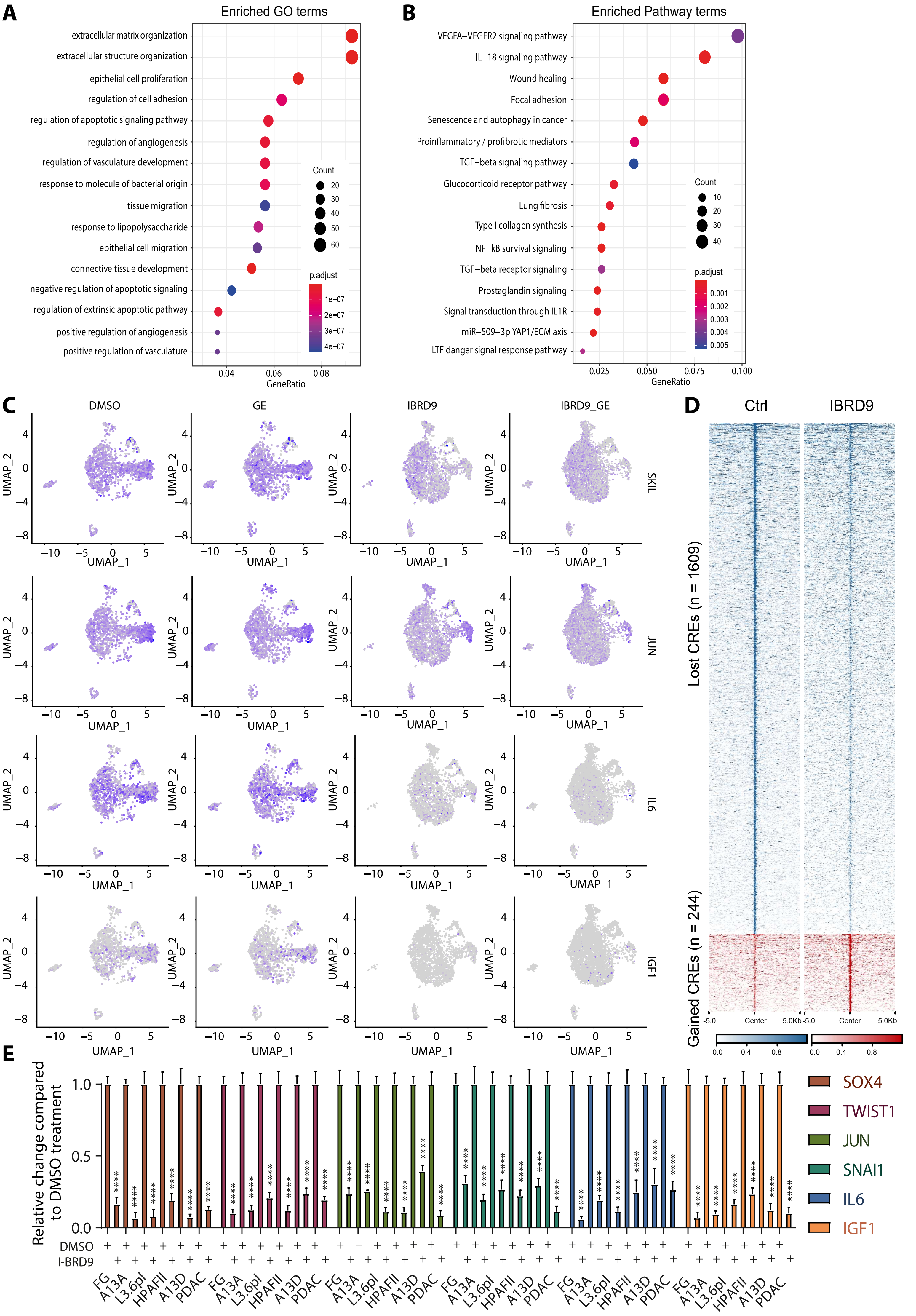
A
Enriched GO terms
extracellular matrix organization
extracellular structure organization
epithelial cell proliferation
regulation of cell adhesion
regulation of apoptotic signaling pathway
regulation of angiogenesis
regulation of vasculature development
response to molecule of bacterial origin
tissue migration
response to lipopolysaccharide
epithelial cell migration
connective tissue development
negative regulation of apoptotic signaling
regulation of extrinsic apoptotic pathway
positive regulation of angiogenesis
positive regulation of vasculature
Count
20
30
40
50
60
p.adjust
1e−07
2e−07
3e−07
4e−07
0.04
0.06
0.08
GeneRatio
B
Enriched Pathway terms
VEGFA−VEGFR2 signaling pathway
IL−18 signaling pathway
Wound healing
Focal adhesion
Senescence and autophagy in cancer
Proinflammatory / profibrotic mediators
TGF−beta signaling pathway
Glucocorticoid receptor pathway
Lung fibrosis
Type I collagen synthesis
NF−kB survival signaling
TGF−beta receptor signaling
Prostaglandin signaling
Signal transduction through IL1R
miR−509−3p YAP1/ECM axis
LTF danger signal response pathway
Count
10
20
30
40
p.adjust
0.001
0.002
0.003
0.004
0.005
0.025
0.050
0.075
0.100
GeneRatio
C
DMSO
GE
IBRD9
IBRD9_GE
SKIL
JUN
IL6
IGF1
UMAP_1
UMAP_2
D
Ctrl
IBRD9
Lost CREs (n = 1609)
Gained CREs (n = 244)
-5.0
Center
5.0Kb
0.0
0.4
0.8
E
Relative change compared to DMSO treatment
1.0
0.5
0.0
DMSO
I-BRD9
FG
A13A
L3.6pl
HPAFII
A13D
PDAC
SOX4
TWIST1
JUN
SNAI1
IL6
IGF1
****

A

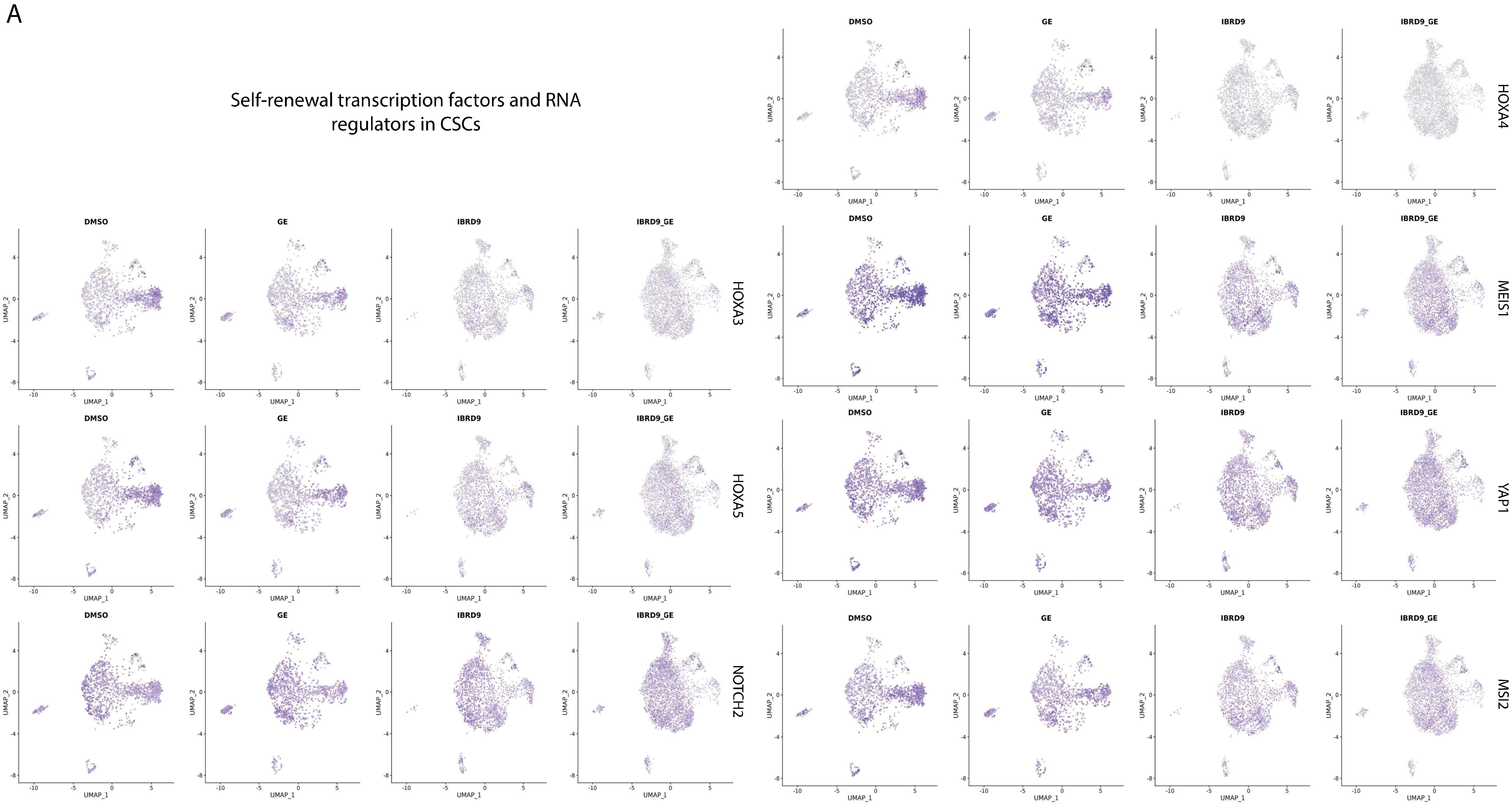
Self-renewal transcription factors and RNA regulators in CSCs
DMSO
GE
IBRD9
IBRD9_GE
HOXA4
HOXA3
MEIS1
HOXA5
YAP1
NOTCH2
MSI2
UMAP_1
UMAP_2

B

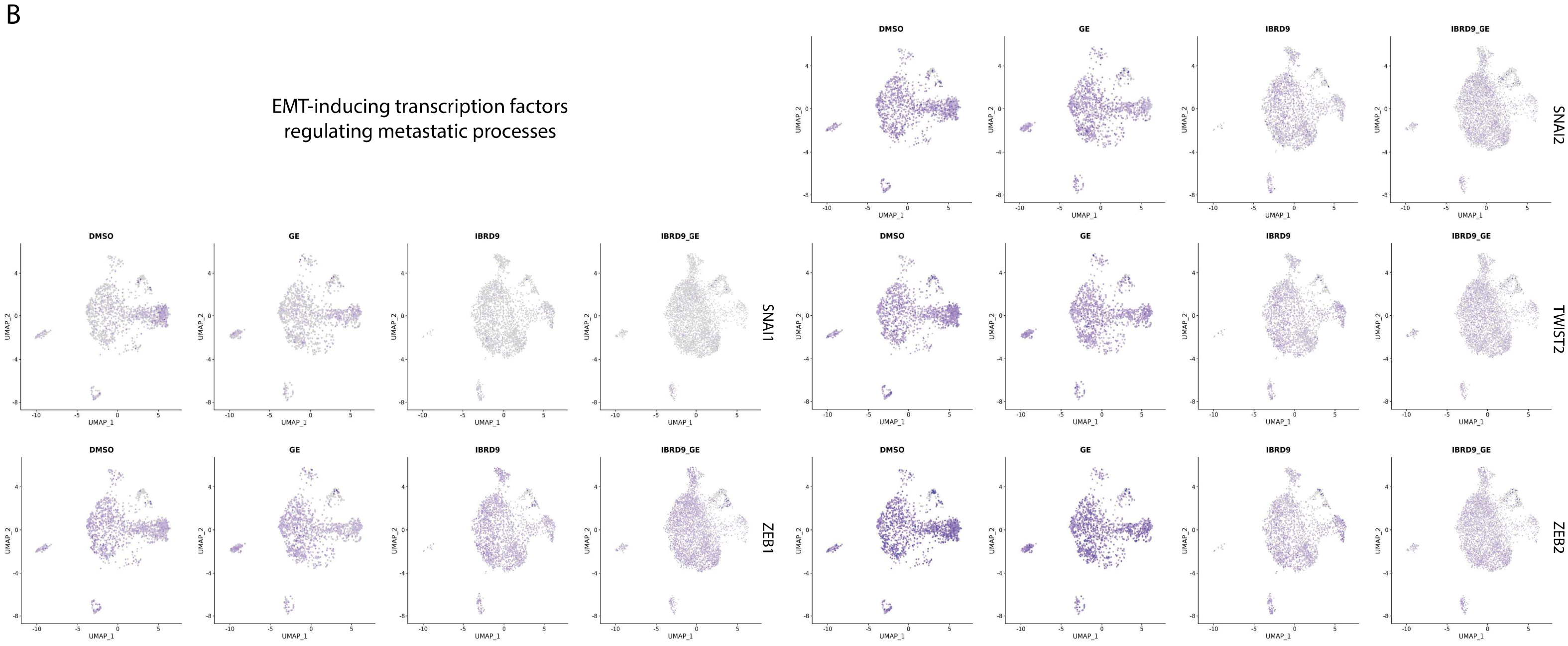
EMT-inducing transcription factors regulating metastatic processes
DMSO
GE
IBRD9
IBRD9_GE
SNAI2
SNAI1
TWIST2
ZEB1
ZEB2
UMAP_1
UMAP_2

C

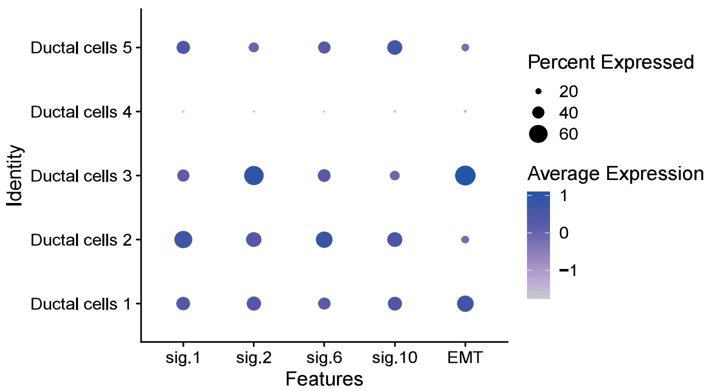
Ductal cells 5
Ductal cells 4
Ductal cells 3
Ductal cells 2
Ductal cells 1
Identity
sig.1
sig.2
sig.6
sig.10
EMT
Features
Percent Expressed
20
40
60
Average Expression
1
0
−1

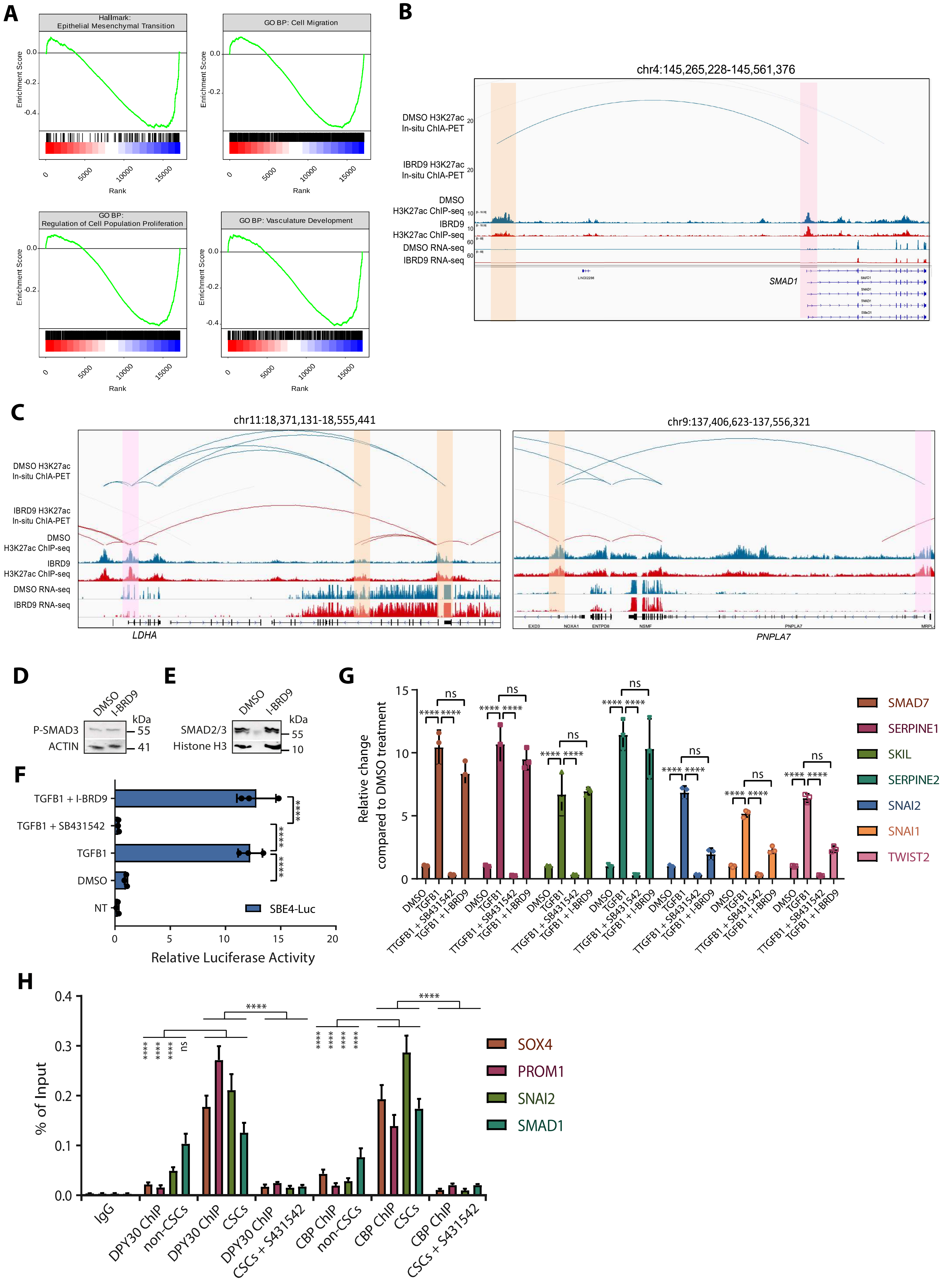
A
Hallmark: Epithelial Mesenchymal Transition
GO BP: Cell Migration
GO BP: Regulation of Cell Population Proliferation
GO BP: Vasculature Development
Enrichment Score
Rank
B
chr4:145,265,228-145,561,376
DMSO H3K27ac In-situ ChIA-PET
IBRD9 H3K27ac In-situ ChIA-PET
DMSO H3K27ac ChIP-seq
IBRD9 H3K27ac ChIP-seq
DMSO RNA-seq
IBRD9 RNA-seq
SMAD1
C
chr11:18,371,131-18,555,441
chr9:137,406,623-137,556,321
LDHA
PNPLA7
D
DMSO
I-BRD9
kDa
P-SMAD3
ACTIN
55
41
E
SMAD2/3
Histone H3
55
10
F
TGFB1 + I-BRD9
TGFB1 + SB431542
TGFB1
DMSO
NT
SBE4-Luc
Relative Luciferase Activity
G
Relative change compared to DMSO treatment
SMAD7
SERPINE1
SKIL
SERPINE2
SNAI2
SNAI1
TWIST2
ns
H
% of Input
IgG
DPY30 ChIP non-CSCs
DPY30 ChIP CSCs
DPY30 ChIP CSCs + S431542
CBP ChIP non-CSCs
CBP ChIP CSCs
CBP ChIP CSCs + S431542
SOX4
PROM1
SNAI2
SMAD1
